# Acceptability and Feasibility of Delivering Pentavalent Vaccines in a Compact, Prefilled, Autodisable Device in Vietnam and Senegal

**DOI:** 10.1371/journal.pone.0132292

**Published:** 2015-07-17

**Authors:** Elise Guillermet, Hamadou M. Dicko, Le Thi Phuong Mai, Mamadou N’Diaye, Fatoumata Hane, Seydina Ousmane Ba, Khadidjatou Gomis, Nguyen Thi Thi Tho, Nguyen Thi Phuong Lien, Phan Dang Than, Tran Van Dinh, Philippe Jaillard, Bradford D. Gessner, Anais Colombini

**Affiliations:** 1 Agence de Médecine Préventive (AMP), Ferney-Voltaire, France; 2 Ministry of Health, National Institute of Hygiene and Epidemiology, Hai Ba Trung, Ha Noi, Viet Nam; 3 Ministry of Health, Fann Résidence, Dakar, Senegal; 4 University of Ziguinchor, Ziguinchor, Senegal; University College Cork, IRELAND

## Abstract

**Background:**

Prefilled syringes are the standard in developed countries but logistic and financial barriers prevent their widespread use in developing countries. The current study evaluated use of a compact, prefilled, autodisable device (CPAD) to deliver pentavalent vaccine by field actors in Senegal and Vietnam.

**Methods:**

We conducted a logistic, programmatic, and anthropological study that included a) interviews of immunization staff at different health system levels and parents attending immunization sessions; b) observation of immunization sessions including CPAD use on oranges; and c) document review.

**Results:**

Respondents perceived that the CPAD would improve safety by being non-reusable and preventing needle and vaccine exposure during preparation. Preparation was considered simple and may reduce immunization time for staff and caretakers. CPAD impact on cold storage requirements depended on the current pentavalent vaccine being used; in both countries, CPAD would reduce the weight and volume of materials and safety boxes thereby potentially improving outreach strategies and waste disposal. CPAD also would reduce stock outages by bundling vaccine and syringes and reduce wastage by using a non-breakable plastic presentation. Respondents also cited potential challenges including ability to distinguish between CPAD and other pharmaceuticals delivered via a similar mechanism (such as contraceptives), safety, and concerns related to design and ease of administration (such as activation, ease of delivery, and needle diameter and length).

**Conclusions:**

Compared to current pentavalent vaccine presentations in Vietnam and Senegal, CPAD technology will address some of the main barriers to vaccination, such as supply chain issues and safety concerns among health workers and families. Most of the challenges we identified can be addressed with health worker training, minor design modifications, and health messaging targeting parents and communities. Potentially the largest remaining barrier is the marginal increase in pentavalent cost – if any – from CPAD use, which we did not assess in our study.

## Introduction

The Global Vaccine Action Plan objectives include strengthening immunization demand, extending benefits to all people, and sustaining program access to predictable and high quality vaccines [1]. One way to support these goals is through use of innovative technologies combined with assessment of their feasibility and acceptability to anticipate and overcome barriers.

Prefilled syringes are the standard in developed countries and have several advantages over vaccine delivery using multi-dose or single dose vials combined with an autodisable single-use syringe. For example, prefilled syringes can increase access to immunization services by simplifying delivery and disposal requirements and thus allowing lay community health workers and midwives to deliver vaccinations. They can reduce vaccine wastage and stock out by avoiding a mismatch between vaccine and injection material. They can also increase injection safety by reducing vaccine handling during the preparation phase.

The primary disadvantage of the prefilled syringes currently used in developed countries is their large volume per dose and thus increased demand on cold chain storage capacity, which in turn has limited their use in developing country settings. This issue has increased in importance with the introduction of new vaccines in the national immunization program in low and middle-income countries, and subsequent requirements for increased investments in costly and difficult to maintain equipment necessary for maintaining end-to-end cold chain and vaccine quality.

**Fig 1:**
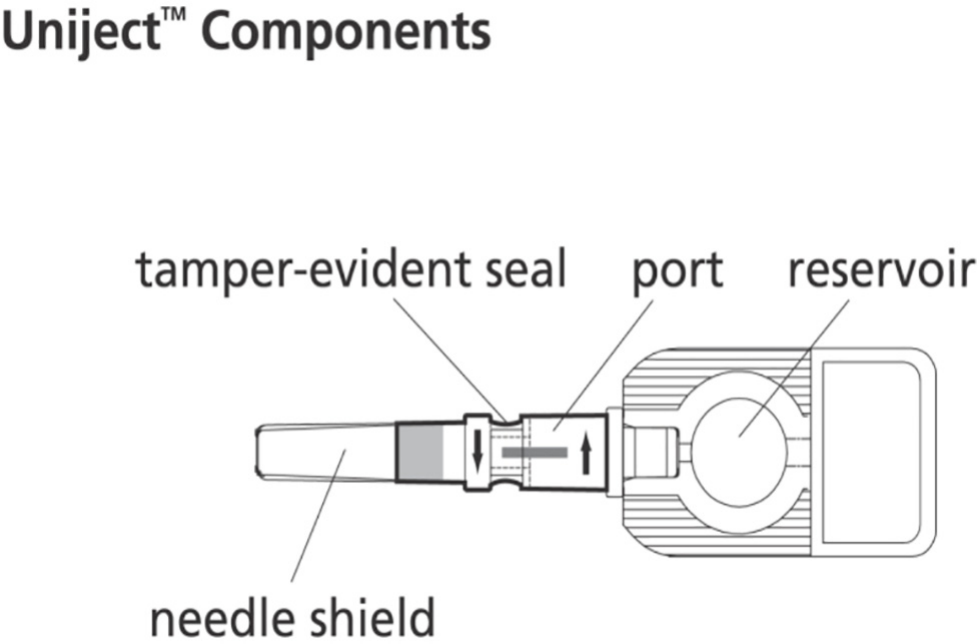
Uniject components.

**Fig 2:**
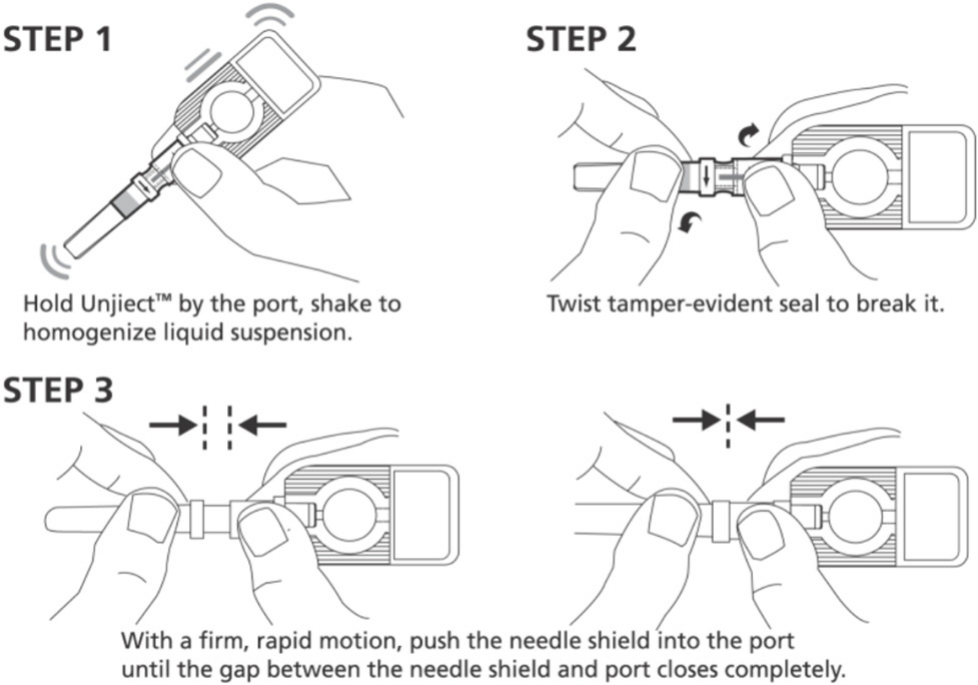
Instructions for the activation and use of the Uniject device.

Uniject–a type of compact, prefilled, autodisable (CPAD) injection system–was originally developed by PATH to overcome barriers to prefilled syringes in developing countries (Figs 1 and 2). Evaluation studies have been conducted for Uniject use during tetanus toxoid campaigns and administration of birth-dose hepatitis B vaccine in routine immunization [2–5]. The current study aimed to assess the acceptability and feasibility by field actors in two countries (Senegal and Vietnam) of using a CPAD injection system to deliver liquid diphtheria-tetanus-pertussis-hepatitis B-*Haemophilus influenzae* type b (DTP-HepB-Hib) pentavalent vaccine developed by Crucell (and recently licensed by the Korean NRA). Formal testing of the technical specifications and engineering of the product was outside the scope of this study.

We chose to work in Senegal and Vietnam in part because of their eligibility for support from Gavi, the Vaccine Alliance. Additionally, while immunization coverage is relatively high in both countries (DTP3 coverage of 80% in Senegal and 93% in Vietnam during 2010), both countries identified specific issues that have prevented coverage improvements such as missed opportunities for immunization, immunization refusal, and difficult to reach populations. This presented the opportunity to assess whether the CPAD device could address some of these issues and thus to improve the likelihood of success should pilot testing subsequently occur. At the time of the study, Senegal and Vietnam used a fully liquid, single-dose pentavalent vaccine with a packed volume varying from 10.3 cm3 to 17.2 cm3 per dose. Both countries already used CPAD-based injection systems for oxytocin delivery, Vietnam for insulin, and Senegal for contraceptives during demonstration projects.

## Materials and Methods

### Site selection

The study teams and national Ministry of Health (MoH) personnel selected two districts in each country (one rural and one urban) that were reasonably accessible for the study team, given budget and timeline limitations. Each site also had strategies in place to access hard-to-reach populations (an outreach strategy in Senegal and social mobilization in Vietnam). Additional criteria included having areas likely to provide a range of attitudes towards vaccines, one district with low and one with high immunization coverage relative to other districts, and a qualitative assessment by national immunization program staff that the districts had at least some occurrence of immunization refusal among target populations. This latter criterion was used so that we could assess the impact of the CPAD injection system on some issues associated with refusal.

In each district, two delivery facilities were selected based on their level of functionality (with one low and one high functioning site), determined by pentavalent coverage and wastage rates. This was assessed in Vietnam using a national coverage survey and in Senegal with input from district medical officers (as a strike made health data unavailable). By partly focusing on facilities with low immunization coverage, the team aimed to assess if CPAD would have a greater benefit in facilities with struggling immunization systems. A second selection criterion was the absence of use of CPAD-based delivery systems for non-vaccine drugs to ensure that this did not bias the perceived utility of CPAD for immunization.

### Feasibility study

To assess the impact on immunization logistics and programmatic issues, we used a structured interview tool to interview and record responses of 29 health workers involved in the national immunization program at central (4), region (7), district (6), and service level (12). The topics covered by the feasibility study included, vaccine and injection device supply chain organization and management; immunization safety and waste management; immunization delivery, vaccine preparation and administration processes; ease of CPAD use; and community involvement in EPI management and communication and social mobilization. Feasibility study data were collected using checklists and tables. Logistics data were analyzed, using the WHO-developed MS Excel immunization logistics planning tool [6]. By entering immunization schedule, vaccine presentation, cold chain equipment capacity, and vaccine distribution and management procedures and practices, this tool provides to immunization logistics managers estimates of vaccine and immunization supply needs and generated waste volume and weight. These results assist in planning the vaccine supply chain at various levels of the health system in the country (cold and dry storage and distribution, and waste management requirements). The Senegal National Immunization Program uses this tool for planning and preparing for new vaccine introduction while the Vietnam National Immunization Program uses a similar tool that was developed locally.

Additionally, we observed healthcare worker immunization practices and their clinic environment and reviewed immunization program documents at the healthcare facility (Table 1). At the healthcare facility delivering immunizations, study staff injected vaccine into oranges to demonstrate CPAD use, as CPAD is not yet licensed for delivery of pentavalent vaccine in the study countries. Following a demonstration, health agents of the eight health posts practiced roughly 10 injections each before their performance was assessed. Data was collated for eight immunization sessions conducted by trained health workers during which 40 pentavalent injections were delivered to oranges using CPAD and 40 using the regular auto disable syringe. We compared the time needed for injection between each group. This outcome included the time for preparation of the injection device, administration of vaccine, and disposal of the used device in a safety box. After practicing, vaccinators were interviewed to determine CPAD acceptability.

**Table 1:** Persons interviewed to assess logistics and programmatic issues with a CPAD system for pentavalent vaccine delivery.

SENEGAL (N = 18 INTERVIEWEES)		VIETNAM (N = 11 INTERVIEWEES)	
Sites	Interviewees	Sites	Interviewees
Central Level	EPI Director and EPI officer in charge of Logistics	Central Level	EPI Director and Logistics Officer
Region 1: Dakar	EPI Focal point and EPI officer in charge of Logistics	Region: North Region	EPI Focal point
District 1.1: Rufisque	District Medical Officer and Pharmacist	Province 1: An Giang	EPI Focal point
Health Post 1.1.1: Sangalkam	Health Post Nurse and Vaccinator	District 1.1: Cho Moi	EPI Focal point
Health Post 1.1.2: Bambilor	Health Post Nurse and Vaccinator	Commune health level 1.1.1: Kien an	EPI Focal point
Region 2: Louga	EPI Focal point and EPI officer in charge of Logistics	Commune health level 1.1.2: KienThanh	EPI Focal point
District 2.1: Linguère	District Medical Officer and EPI officer in charge of Logistics	Province 2: Dien Bien	EPI Focal point
Health Post 2.1.1: Linguère	Health Post Nurse and Vaccinator	District 2.1: Tp. Dien Bien	EPI Focal point
Health Post 2.1.2: Barkedji	Health Post Nurse and Vaccinator	Commune health level 2.1.1: Ta Leng	EPI Focal point
		Commune health level 2.1.2: Nam Thanh	EPI Focal point

### Acceptability study

To measure the acceptability of CPAD technology, we conducted semi-structured interviews with lay health workers and formal health workers at healthcare facilities, representatives of health professional societies at central level, and EPI representatives at central and service levels. We also interviewed caretakers and community members in the selected districts with different characteristics using a purposive sample based on job function, ethnicity, gender and known attitudes towards vaccine. A purposive rather than a random sample was used to ensure inclusion of people with different characteristics that migh influence attitudes towards the CPAD injection system.

In Senegal, we conducted five focus groups with 20 caretakers (two to seven persons per group) and 49 interviews with caretakers (n = 15), community representatives (n = 9) and immunization stakeholders (n = 25). In Vietnam, we conducted four focus groups with 15 village health workers (three to six persons per group) and 72 interviews with immunization stakeholders (n = 17), community representatives (n = 16), and caretakers (n = 39) (Table 2). Immunization stakeholders including lay and formal health workers were identified purposively based on their roles in delivering immunization. Caretakers in Senegal were selected during immunization sessions based on having representation of people with and without vaccine hesitancy (based on health worker assessment). In Vietnam, service level health workers in health facilities identified all caretakers in advance by to facilitate scheduling interviews. (Table 3)

**Table 2:** Distribution of interviewees by country, sites and type for an anthropological assessment of a compact, prefilled, autodisable device (CPAD) system for delivery of pentavalent vaccine.

Country	Caretakers	Community representatives	Immunization stakeholders	Total
**Senegal**				
Site 1	17	4	13	34
Site 2	18	5	12	35
Country total	35	9	25	**69**
**Vietnam**				
Site 1	19	8	17	44
Site 2	20	8	15	43
Country total	39	16	32	**87**
**Both countries**	**74**	**25**	**57**	**156**

**Table 3:** Categorization of interviewed caretakers by attitudes regarding national immunization program infant immunization in Senegal and Vietnam.

	Accepts immunization (regardless of doubts)	Accepts immunization but missed opportunities (e.g., lack of vaccination card, lack of vaccines)	Accepts immunization but may delay (e.g., time constraints, false indications)	Refusal of some vaccines	Refusal of all vaccines (e.g., pain due to injection, inject safety concerns)	Total
**Number in Senegal**	20	1	8	3	3	35
**Number in Vietnam**	20	13	6	0	0	39
**TOTAL**	**40**	**14**	**14**	**3**	**3**	**74**

Focus groups and interviews followed semi-structured guidelines. Key questions were developed by the study team based on an informal literature review of immunization and injection perceptions and practices as well as health system organization, including that of lay health workers [4,7–18]. EPI representatives of both countries reviewed guidelines and provided modifications, following which pre-testing occurred in Dakar and Hanoi. All questions in the guidelines were used for interviews and additional topics were discussed as they emerged in specific interviews to capture the full range of views.

A study team member demonstrated CPAD device use to all interviewees. Study subjects were free to manipulate the device. Vaccinators that participated in the feasibility study were included in the acceptability study.

Interviews were recorded, transcribed and translated. Responses were coded based upon pre-selected themes identified in the guidelines. Responses outside of those included in the guidelines were also coded. Quotations from different interviews regarding the same topic were compiled and compared to identify all existing viewpoints. For each country, two study team members were involved in this latter process, one from the national team and one international investigator and final results were determined by consensus.

We did not use a statistically representative sample of respondents but rather sought to obtain the range of viewpoints existing within the community. Consequently, we could not quantify the relative prevalence or importance of different viewpoints, although at various points we indicate general response frequency (e.g., “some” or “many”) for additional context.

### Ethical approval

In Senegal, the Ethical Review Board of the Ministry of Health and Social Action reviewed and approved the study protocol and staff from this group supervised implementation of the study in the field to verify the informed consent process and that the CPAD device was not used on human subjects. In Vietnam, the Institutional Review Board of the National Institute of Hygiene and Epidemiology reviewed and approved the study protocol. All interviewees in both countries provided written, informed consent using a form approved by the study Institutional Review Boards. Study data were anonymized to maintain confidentiality.

## Results

### Main advantages

#### Feasibility

The feasibility assessment evaluated CPAD’s projected impact vaccine supply chain organization and management in comparison with existing vaccine presentations in the two study countries. The marginal change in cold chain requirements that would result from CPAD use depended on the presentation and formulation of the pentavalent vaccine currently in use, as well as the immunization schedule and vaccine procurement and stock management practices. Senegal uses single dose pentavalent vaccine from three different manufacturers, with an average volume size per dose of 17.2 cm^3^, which is higher than the 13.7 cm^3^ of CPAD. Consequently, the overall cold chain requirement at the service delivery level for pentavalent vaccine would decrease 12% per fully immunized child (FIC) from 89.3 cm^3^ with current ADSs to 78.9 cm^3^ with CPAD. Vietnam, uses only single-dose pentavalent provided by Crucell with a volume per dose of 10.2 cm^3^, which is lower than the 13.7 cm^3^ for CPAD. Consequently, the cold chain volume would increase 5% from 100.9 cm^3^ per FIC with current ADSs to 106.2 cm^3^ with CPAD.

The volume and weight of injection material (i.e., syringe, needle, and safety box which is recommended by WHO for injection safety purposes), impact the feasibility of outreach immunization activities, since bicycle and motorbike are used to carry both vaccinators and material to conduct immunization sessions. In Senegal, injection material volume for pentavalent vaccine administration was 570.3 cm^3^ per fully immunized child using a traditional ADS and 391.8 cm^3^ per fully immunized child with CPAD, a reduction of 31%; injection material weight was 70.4g with ADS and 56.5g per fully immunized child with CPAD, a reduction of 20%. In Vietnam CPAD use resulted in a volume reduction of 17% compared to ADS (from 1045.6 to 867.1 cm^3^ per fully immunized child) and a weight reduction of 11% (from 130.2g to 116.3g per fully immunized child). These reductions occurred due to lower syringe volume and weight.

Many vaccinators and program managers noted that CPAD might reduce stock-outs (and consequently reduce low coverage or missed opportunities) because it integrates vaccine, syringe, and needle. They also reported that its lower weight and volume could improve outreach or catch-up campaign efforts by reducing supply needs.

During the injection simulation, we observed that the average time per injection in an orange decreased from 46 seconds with ADS to 29 seconds with CPAD (maximum times were, respectively, 65 and 48 seconds). In Senegal CPAD reduced administration time by 27–35% and in Vietnam by 40–61%. Timing for both ADS and CPAD included all technical steps from the point at which the child was in front of the vaccinator to device disposal.

#### Acceptability

Many interviewees among all categories reported that CPAD seemed easy to use, especially for preparation (Table 4, S1 and S2 Data). Participants reported pre-filling might improve efficacy in general; health workers expanded on this idea by indicating CPAD would ensure an accurate dose compared to existing autodisable syringes (ADS) since the latter can interrupt vaccine delivery due to the blocking mechanism.

**Table 4:** Summary of advantages and challenges according to interviewees.

Characteristics	Advantages	Challenges
**Design**	1) Looks modern (all categories of interviewees); 2) The device is shorter, which might be easier to use (all categories of interviewees in Senegal); 3) The needle looks shorter, which is better for avoiding pain (all categories of interviewees in the urban area of Senegal); 4) Reduces children’s fear (all categories of interviewees in Senegal and Vietnam).	1) Shorter needle is less effective (many caretakers in the remote area in Senegal); 2) The needle looks bigger and might be more painful for infants (many caretakers and some health workers in Vietnam); 3) Font size on the label is difficult to read (observed).
**Safety**	1) Non-reusable (some health workers and caretakers in both countries); 2) No exposure of needle to air or dust during activation (many health workers in both countries); 3) No damage of needle during vial opening (some health workers in both countries); 4) No exposure of vaccine during preparation (some health workers in both countries); 5) Needle under cover, which avoids risk of contact (some health workers in both countries).	1) Not retractable like ADS and nurse representatives have asked for safer devices (two health workers in Senegal); 2) Doubts about interference of between CPAD plastic container and vaccine (some health workers in both countries); 3) Perceived risk of freezing (some health workers in Senegal); Perceived risk of ‘breaking bones’ because of needle’s length (many caretakers and health workers in Vietnam); 4) Perceived risk of needle breaking in the arm or leg of restless children as the device is thin (some caretakers and some health workers in Vietnam); 5) Tamper seal might fail, with risk of skin contact (some health workers in both countries).
**Ease of use**	1) Simplicity of preparation (all categories of interviewees in both countries); 2) Timesaving for health staff and caretakers, which is useful for avoiding missed opportunities and local conflicts and reduceing children’s fear (some health workers and some caretakers in both countries).	1) Difficult to activate rotary movement since it needs strength and hurts hand (many health workers in Vietnam); 2) The movement to drill the vaccine container needs strength (some health workers in Vietnam); 3) Difficult to squeeze the whole vaccine (some health workers in both countries), which may also prolong injection and pain (two health workers in both countries); 4) Handle part is too tight to maintain the device in the arm/leg (some health workers in Vietnam); 5) Failure during injection may increase vaccinator risk, risk of missed opportunities, and vaccine wastage (some health workers in both countries).
**Dose**	Dosing more accurate because industrial measurement safer than human one (some health workers in Vietnam).	1) Perception that quantity not sufficient (some health workers and caretakers in both countries); 2) Perception that needed vaccine left in vial (some health workers in both countries).
**Supply chain**	Reduced stock outages (and hence reduction in missed opportunities) due to absence of a blocking mechanism such as in ADS, the lack of risk of glass vials breaking, and the bundling of vaccine and syringes, which eliminates the chance of a shortage of one or the other; Reduction of total injection material (vaccines, syringes, safety box) volume and weight	1) Perceived increase cold chain volume needed (some health workers in both countries); 2) Need for bigger cold boxes (some health workers in both countries).

Respondents perceived that CPAD improved safety in several ways. This injection system eliminates vaccine exposure during syringe preparation, which ensures vaccine and needle sterility. The activation mechanism allows the needle to remain covered until injection, and the device’s packaging prevents dust and air exposure (reported by many health workers and some caretakers). The risk of needle damage during vial opening (some health workers) is reduced as well as the risk of needle-associated injuries during syringe preparation (reported by some health workers). Finally, respondents reported that CPAD’s shorter device may decrease anxiety among vaccinees compared to standard syringes and make vaccine delivery easier for anxious caregivers and children (reported by many health workers, some community representatives and some caretakers).

### Main challenges

Immunization stakeholders who had used the CPAD device–including vaccinators who participated in the feasibility study–indicated that twisting the tamper-evidence seal to break it (step 2) and pushing the needle into the port (step 3, activation) may be difficult (Fig 2), a difficulty that decreased with practice (Table 4, S1 and S2 Data). The study team also observed this during the manipulation of CPAD or administration in oranges. Climate characteristics might be one of the causes of the difficulties observed. For example, in Vietnam (with a humid climate) the primary difficulty identified was the twisting motion to break the tamper-evidence seal, while in Senegal the primary difficulty was the push needed for system activation. In the event of activation failure, the vaccinator would have to repeat the procedure, which many vaccinators in Senegal worried might impact staff or equipment credibility. Finally, some vaccinators were concerned that the twisting motion could cause irritation or inflammation of vaccinators’ hands after repetition during an immunization session.

Many vaccinators noted difficulty conducting the proper motion during injection. Some vaccinators found the port, which is shorter than that for ADSs, difficult to hold; this may have occurred because ADSs are designed for delivery at a 45-degree angle compared to 90 degrees for CPAD-based delivery systems. Some vaccinators noted that reservoir flexibility could lead to difficulty maintaining the needle in the thigh, especially among restless children. Vaccinators in Vietnam perceived that this might cause separation of the needle from the port during injection, a perception possibly related to the common use in Vietnam of syringes with detachable needles. Some vaccinators indicated that an undesirable amount of strength is necessary to inject vaccine. Finally, some vaccinators wondered about the significance of the residual dose left in the reservoir after injection, specifically whether the appropriate dose was injected and by extension whether the injection would be effective; this may have occurred due to lack of a final stopping point for CPAD, in contrast to a definitive endpoint for ADSs.

Interviewees expressed concern regarding the non-sterile nature of the tamper-evidence seal and risk for infection if the seal was not properly broken and then came into contact with a child’s skin. Similarly, interviewees expressed concern about the risk of skin abrasion of vaccinees due to friction from a residual piece of the tamper-evidence seal.

Health workers questioned whether CPAD’s plastic (versus glass) reservoir and box would a) protect vaccine from the risk of freezing in the cold chain to the same degree that glass does or b) interfere with vaccine content.

CPAD has a 23-gauge 1 inch (25 mm) needle, the WHO’s recommended standard length for intramuscular injection at 45 degrees [19]. Respondents in different sites perceived needle length and gauge differently. In Vietnam, many interviewees (including caretakers and health workers but excepting two experienced vaccinators) perceived the CPAD needle as longer and bigger than that for ADSs (even though objectively only the gauge of CPAD is bigger than for ADS). Some of these interviewees said that needle length standards are defined for children of high income countries and may be inappropriately long for Vietnamese children, increasing the risk of bone, muscle and nerve damage. In Senegal, the needle and syringe lengths were associated with anticipated pain and vaccine efficacy by many caretakers and community representatives. In one of two Senegalese sites, the CPAD needle was perceived as too short, and thus potentially less efficacious. In contrast to Vietnam, many respondents among all categories did not perceive that injury from vaccine was associated with needle length but rather with vaccinator skills, including the ability to serve children with a “light hand”.

At central and operational levels, some immunization stakeholders raised the potential risk of confusion with medicines already delivered via CPAD such as contraceptives, with a subsequent risk of adverse rumors regarding CPAD content. Also, health workers in Senegal indicated that if immunization staff and recipients think that CPAD presentations are produced at low costs solely for resource-poor settings, these stakeholders might have reduced confidence in the device and its contents.

## Discussion

We conducted an evaluation in two developing countries of CPAD, an existing technology that has been adapted for delivery of pentavalent vaccine. Our review of current immunization practice, and results of stakeholder interviews, showed that CPAD use could lead to several advantages over existing ADSs. Some stakeholders perceived vaccine delivered through CPAD to have improved safety and efficacy in some respects. For several immunization logistics and supply chain challenges, CPAD may decrease missed immunization opportunities. Bundling vaccines and syringes will prevent unbalanced stock-outs of one or the other component. This feature along with decreased weight and volume should improve outreach campaigns. The single dose presentation should reduce vaccine wastage associated with multi-dose vials. Lastly, similar to previous studies [20] we found that simulated CPAD use reduced the average time per injection. However, the usefulness of a reduction in average injection time of 17 seconds is not clear, since injection time represents a small fraction of the total time required to vaccinate a child. Additionally, the marginal economic value of CPAD compared to existing presentations will depend on additional factors such as vaccine costs in the different presentations as well as cold chain requirements [21,22].

In theory CPAD use also could reduce missed opportunities if its ease of use led government bodies that sanction vaccinators to be more likely to approve lay vaccinators for immunization delivery, since this in turn would increase the pool of vaccinators. Previous studies have indicated overall community and health worker acceptance of lay vaccinators [23], including when using CPAD to deliver vaccines [24]. However, some of the responses provided by informants suggested that ease of vaccine delivery was a less important factor than injection safety for lay vaccinator acceptance, since safety is considered the vaccinator’s responsibility and deficits in this area could lead to loss of credibility.

Despite these advantages, we identified several key challenges for CPAD acceptability that should be addressed before widespread use. Interviewees in both countries had concerns regarding injection safety with CPAD. In Senegal safety concerns were linked to the skill of vaccinators, an issue that presumably can be solved through training and communication between vaccinators and patients. In Vietnam, interviewees had concerns regarding needle length and gauge, potential pain or injury, and the risk of separation of the needle from the syringe or needle breakage. We could not assess if these concerns derived from previous experience of ADSs or detachable syringes (used in Vietnam).

Regardless, our results suggest that the needle size in new technologies may need to be adapted to local settings. The 23-gauge by 25 mm size of CPAD is that recommended by the US Centers for Disease Control and Prevention and the American Academy of Pediatrics, as is the needle insertion angle [25,26]. This recommendation exists in part because of reports that 23-gauge 25 mm needles lead to substantially less reactogenicity than 25-gauge 16 mm needles for delivery of DPT and Hib conjugate vaccines [27], although this finding may vary with insertion angle (45 or 90 degrees) [19]. In sum, global stakeholders and manufacturers should consider the local demand to adapt needle size to local contexts, including child weight-for-height, injection techniques, and previous experience of AEFI occurrence resulting from injection technique; the latter two issues could be assessed with surveys in target countries.

We found several issues related to packaging, such as the potential confusion of vaccines with other pharmaceutical products already delivered through a CPAD device. To avoid confusion with other products delivered in similar delivery systems, the manufacturer should consider use of more legible labels and one or more distinguishing characteristics based on labels, colors or shape. This should reduce the risk of inadvertent delivery of an incorrect pharmaceutical or vaccine and reduce risk of rumors based on associating vaccines with products such as contraceptives. Package design should take into account the high level of illiteracy in many countries; for example, distinctions in color or shape may prove more useful than relying solely on text labels.

We found evidence that vaccines perceived as produced particularly for low-income settings may have reduced acceptability among some populations, based on a perceived inferiority in performance. This result occurred despite the opinion of informants that CPAD is more modern and safer. This issue may be amplified by messaging on CPAD’s ease of use, and its use by lay vaccinators. This ambiguous perception of innovation might be addressed by messaging during parental information and health worker training sessions.

Informants reported difficulty in breaking the tamper-evidence seal and risk of skin abrasion of vaccinees due to friction from a residual piece of the tamper-evidence seal. The manufacturer has addressed both of these issues through product re-design based on our study results, so these issues should no longer exist. Additionally, informants had concerns about whether the plastic reservoir and box protect vaccine from the risk of freezing in the cold chain to the same degree that glass does and whether plastic will interfere with the vaccine. We did not evaluate the validity of these concerns, but if plastic does not increase risk over glass, these issues suggest a topic area for product messaging rather than a need for re-design.

The results of the current study are a first step and should be followed by small-scale demonstration projects of CPAD integration into actual immunization programs. These projects should include replication of our methods (including assessment of the concerns we identified) plus the addition of a quantitative component. A quantitative study would allow for determination of the relative importance of different issues that remain with CPAD and would extend the ability of immunization programs to assess the relative advantages of CPAD use compared to other presentations. Lastly, future studies should assess the cost and cost-benefit of CPAD compared to traditional ADS.

## Conclusions

Delivery of pentavalent vaccine using CPAD has the potential to address some of the main barriers to vaccination, such as supply chain issues and safety concerns among health workers and families. Several issues remain to be addressed, mostly through health worker training, minor design modifications, and health messaging targeting parents and communities. Key communication and education topics will need to focus on CPAD usability, safety, and efficacy. The long term benefit of CPAD for pentavalent vaccine delivery will depend on the speed with which manufactures can make available needle-free delivery systems [28,29] that are safe, reliable, and accepted by the health workers and vaccine recipients. Introduction of pentavalent vaccine on a wider scale should occur with feasibility and acceptability evaluations in additional settings with a focus on the issues identified in the current study and a proactive approach to incorporating community perceptions of appropriate immunization systems.

## Supporting Information

S1 Data Acceptability study results for Senegal. (DOCX)

S2 Data Acceptability study results for Vietnam. (DOCX)
