# Supplementary material for: Acceptability and Feasibility of Delivering Pentavalent Vaccines in a Compact, Prefilled, Autodisable Device in Vietnam and Senegal
Source: PLoS One. 2015 Jul 17;10(7):e0132292. doi: 10.1371/journal.pone.0132292 (PMC4506041; doi:10.1371/journal.pone.0132292)
Supplement: S1 Data — (DOCX) [file pone.0132292.s001.docx]

# Supplementary file 1: Acceptability study results for Senegal

# Interviewee viewpoints of Uniject^TM^ in Senegal

## Uniject^TM^ as an injection technique

Three attitudes towards the Uniject^TM^ device were observed: i) acceptance, by families who accept vaccines and those who have “given up” on vaccination, but believe in the efficacy of injections, and most health staff, ii) neither acceptance nor refusal, among some health staff with fears about user-friendliness of Uniject^TM^ and needs for training (in this case, the feasibility of using Uniject^TM^ is the condition for its acceptance), iii) refusal, among interviewees who generally oppose vaccines.

## Findings of feasibility / acceptability studies

Responses are summarized by topic: design, safety, efficacy, ease-of-use, dosage, and supply chain (regarding Uniject^TM^ device for pentavalent vaccine).

*Design*

- Appearance: All interviewees spontaneously said that the device looks modern, well designed, and attractive. For some of them, Uniject^TM^ is apparently perceived as more effective thanks to its physical appearance.
- Length of the whole syringe: Some vaccinators and parents noted that the syringe is shorter than that of an ADS. For one of them, the shorter length would reduce the pain for children:
  - - *“The needle is thinner and there is not the same tube. With the other, it takes time to support and bring out the product. It hits the child. With this device, it should be faster. The child will not have time to feel the injection and then it is already finished.” (Caretaker of six children, C3)*

However, one vaccinator had doubts about the ease-of-use of Uniject^TM^ because of its shorter length.

- Length of the needle: The majority of interviewees, among all categories, shared the first impression that needles are shorter (even if this is not the case) and painless. For some of the interviewees, this characteristic was an improvement:
  - - *“The needle seems smaller which is important for mothers who often say that the needles we use are too big for their children.” (Health worker at operational level, IS 19)*

However, for other caretakers the perceived needle length is a disadvantage, as they do not believe that a shorter needle can have the same efficacy as longer ones:

- - - *“(One mother) I do not know if this is a true vaccine so it's fine! I prefer syringes and needles that are currently used. - (Another) It is small. Is it effective? It's too small.” (Two mothers with attitude of vaccine acceptance, during a focus group in a remote area)*
- Labeling: Although only one vaccinator expressed difficulty reading the label, the study team observed that the font size was uncomfortable for many vaccinators^[[1]](#footnote-1)^.

*Safety*

- For health staff/vaccinators: They firstly expressed their trust in non-reusable syringes because of blood-borne diseases. But some of them also pointed to risks if they have to recap the syringe, notably after failing to activate Uniject^TM^^[[2]](#footnote-2)^. Moreover, one nurse who is also a central representative of nurses, expressed the need for retractable syringes to protect vaccinators:
  - - *“Yet for immunization, we have safe syringes for children with ADS, but no innovation for vaccinators! This device is good but we need a retractable syringe.” (Immunization stakeholders, IS5-6)*
- For parents (regarding their children) issues raised included the following:
- Blood-borne diseases: Some parents (2/38; middle-class and with a role of informal practitioner) expressed trust in non-reusable syringes because of blood-borne diseases. One parent said that Uniject^TM^ as a prefilled syringe could provide more evidence of first usage regarding the blister’s appearance after squeezing.
- Adverse events following injection (AEFIs): Both parents and health staff raised the issue of AEFIs, expressing trust in Uniject^TM^ in comparison to ADS because i) AEFIs are partly attributed to external particles like microbes or dust and may be caught during vaccine aspiration, and ii) AEFIs are also attributed to needle damage during vial opening, a problem that is avoided with Uniject^TM^:
  - - *“It seems safer than ADS. You cannot draw microbes. And you avoid dust as well.” (Caretaker with background in health studies, C2)*
- Container material and storage conditions (risk of freezing): Few parents (2/38) expressed doubts about the negative effects of container material on vaccine content in comparison to glass and solid plastic of ADSs. In regards to storage conditions, some immunization stakeholders expressed doubts about the capacity of the container’s material to adequately insulate vaccine, notably when placed inside a freezer.

*Efficacy*

- Except families that oppose all vaccines, there was no doubt expressed about the efficacy of pentavalent content
- Pain: However, respondents indicated that vaccine itself was particularly painful, because it delivers several antigens at once. One graduated health worker shared a common fear with mothers: using several vaccines at once (i.e., pentavalent vaccine) it is too much for children.
- Use of Hep B: Two immunization stakeholders expressed doubts about the political choice of using hepatitis B vaccine in pentavalent rather than in birth-dose vaccine. International protocols regarding delivery of multiple antigens simultaneously are not well known at the operational level. These immunization stakeholders criticized the cost of the birth dose of vaccine for at-risk families. Considering this issue, one of these interviewees concluded that improving devices to address organizational and logistical concerns is often useful but can create public health problems.

*Ease-of-use*

- Activation: Families and community members expressed that activation would be easier for vaccinators. However, after demonstration and testing, vaccinators expressed doubts about ease-of-use, especially regarding rotation (i.e., step 2: “twist tamper-evident seal to break it”) and pushing the needle cap sufficiently far to engage the liquid vaccine (i.e., step 3: “with a firm, rapid motion, push the needle shield into the port until the gap between the needle shield and port closes completely”).
- Risks: Interviewees expressed potential risks including failure to deliver the vaccine; more pain for children; due to the need to inject twice; and needle exposure for vaccinators if they have to recap after the first injection to engage the vaccine. This last action was presented as a necessity to avoid vaccine wastage.
- Injection: One vaccinator described the difficulty of having sufficient pressure to expel the whole quantity of vaccine:
  - - *“It's solid but it seems that the device softens when pressing to expel the vaccine. It is like we can’t use the whole dose. The ease of use might be improved.” (Immunization stakeholder IS5)*

*Dosage:*

- Vaccine dose: The prefilled device is perceived as a guarantee of appropriate vaccine dose for the different categories of interviewees, notably in comparison with ADS:
  - - *“Some vaccinators charge the syringe first. Then they are distracted, they drop off the syringe and the dose is not completed. With UnijectTM it would not be a problem.” (Caretaker with a background in health studies, C2)*
- Vial doses: Some interviewees expressed doubts about content dosage, and wanted to make sure that content was dispensed in the appropriate quantity
- Residual vaccine: Vaccinators observed that not all vaccine was issued during immunization (residual dose left in vial, which is what the manufacturer wants). They asked if the amount of additional vaccine ensures that the right quantity was delivered.

*Supply chain*

Additional stated advantages concern the supply-side, namely safety/storage boxes and ease of transport, given the lighter weight:

- Safety boxes: According to interviewees (vaccinators, garbage managers), UnijectTM devices require less safety boxes because they are smaller than AD syringes
- Storage boxes: According to interviewees, the combination of syringes and vaccines would facilitate the storage and transport of supplies by avoiding the duplication of boxes (one for syringes and one for vials)
- Lighter weight: Graduated health workers and lay health workers stated that the lighter weight of the UnijectTM device may be a solution to current difficulties in outreach strategies related, among others, to the weight of supplies. As such, the UnijectTM device would improve their work conditions.
  - - *“It might be easier to bring vaccines for outreach strategy or campaigns. We bring the cold boxes with the sheets for monitoring and it is heavy. These small boxes seem practical.” (Health worker at the operational level, IS21)*

Main findings of “Uniject^TM^ device advantages and disadvantages” (Senegal)

| **Characteristics** | **Pros** | **Cons** |
| --- | --- | --- |
| **Design** | Looks modern; the device is shorter and the needle looks shorter (perceptions of some vaccinators and most of caretakers): better to avoid pain (urban area) | Shorter needle less effective (perceptions of caretakers in remote area); font size on the label difficult to read |
| **Safety** | Non-reusable; no exposure of needle to air or dust during activation; no damage of needle during vial opening | Like ADS syringes, Uniject^TM^ is not retractable (which is expected by central representatives of nurses for their safety); doubts about effect of container material on vaccine contents; doubts about risk of freezing |
| **Efficacy** |  | Fear of strength of several antigens; doubts about prevention strategy for hepatitis (for penta in general) |
| **Ease-of-use** | Simplicity and time-saving for preparation | Activation needs strength: if misused, there were different risks notably for vaccinators safety, risk of missed opportunities and vaccine wastage; not easy to inject vaccine |
| **Dosage** | Industrial measure can be stated more clearly than human one | Doubts about quantity and residual dose |
| **Supply chain** | Reduction of material and weight: vaccines and syringes and safety boxes (lighter weight a particular benefit for outreach strategies); integrated plastic labels avoid erasure; limited wastage of syringes and vaccines in comparison to ADS; easier cold box storage.  Lightness useful for outreach strategies.  Elimination of errors in calculating requirements of vaccine and syringes. |  |

# Current constraints of vaccination during routine and outreach strategies from the perspective of interviewees in Senegal

## Immunization stakeholder viewpoints: Challenges

This section provides an overview of immunization stakeholder viewpoints, categorized as follows: general lack of tools and human resources; lack of supplies; constraints of mobile and outreach strategies; and refusal attitudes.

*General lack of tools and human resources*

Immunization stakeholders expressed a lack of human resources and means to conduct their activities. Three different strategies to overcome this difficulty were observed:

1. Involvement of lay health workers to provide injections^[[3]](#footnote-3)^:
   - - *“There was no recruitment of graduates for 10 years. Imagine! The lay health workers have saved the health system”. (Immunization stakeholder 5-6*^^[[4]](#footnote-4)^^*)*
2. Data strike: Some Senegalese health worker syndicates led a “data strike” for two years to protest their work conditions. This led to an observed lack of data on immunization activities at central level and even in some health facilities where data reporting was interrupted. This contributed to difficulty determining which populations had and had not been vaccinated.
3. Immunization strike: In addition, an immunization strike was conducted in some areas with the following consequences:

- Interruption of vaccine delivery in some public health facilities:
  - - *“We took the vaccination just a week ago, after two years of interruption. We protested against what we call the "virtual districts." The Ministry of Health split one district into four districts. That does not make sense! Health facilities are not sufficiently equipped to become districts. It is an aberration”. (Health worker IS24)*
- Saturation of health facilities, with no available vaccines in some areas and no outreach strategy:
  - - *“Some nurses boycott immunization. They refuse to conduct this activity. Women chose to come here. This is why we have to vaccinate every day. To protest their situation, nurses do not vaccinate. They say that there is no equipment. So for two years, no vaccine was delivered and there is no outreach strategy.” (Health worker IS15)*
- Temporary collaboration between the district and private practices in the area. This collaboration was interrupted after the strike ended. This practice consisted of the use of EPI vaccines in private structures to replace public health facilities. Consequently, families had to pay for vaccines:
  - - *“I give around 38 vaccinations per month at a cost of 1000 CFA (Senegalese francs) per vaccination. The health center sent me vaccines. I placed orders on the basis of needs and the health center provided the vaccines. I sent a quarterly report to the health center.” (Health worker IS22, in a private practice)*

*Lack of supplies*

Health workers at operational level had various explanations/comments regarding the lack of supplies including vaccines and ADSs. Main observations included the following:

- Poor estimation of vaccine dose needs due to a lack of available population survey data, and national and transnational migrations:
  - - *“Officially, we have four villages that use the health center. But we have more than100% coverage as we have mothers coming from other areas. With floods and new comers in the periphery who come from other regions or countries, I can’t know how many children we have to vaccinate.” (IS7, Health worker at operational level)*
- Vaccine wastage due to technical issues (e.g., improper functioning of ADSs such as blocking or syringe damage):
  - - *“We often have stock-outs of ADSs because of the use of several syringes to achieve the delivery of the right dose. People are forced to buy syringes (…) and with the rubber stoppers on the vials, syringes are damaged. You end up having to use two syringes and the vaccine is lost as they are ADSs.” (Health worker at operational level, IS7)*

Two strategies were described to limit the risk of lack of supplies:

- Having health facility management committees or families buy syringes. No families described this necessity, while some committee managers denounced the heavy financial burden for the committee and the risk of missed opportunities when asking families to pay for syringes.
- Using single-dose vials. In some health facilities, staff described how when using multi-dose vials they waited for enough children to be present (10 or 20) before opening a vial. This led to missed opportunities if families had to come back for another session.
  - - *"The difficulty is that the health facility is not quite crowded and to give the vaccine requires a bottle of 10or 20doses (like the pentavalent vaccine in the past)… Sometimes there are not enough children. So we did two doses per child but rarely the three doses. For people it is a waste of time with three hours of walking round trip. And laborers must work in the fields." (Health worker at the operational level, IS10)*

*Constraints of mobile and outreach strategies*

In health facilities, outreach strategies and sometimes campaigns are considered to be too difficult for various reasons:

- Lack of financial support for outreach strategies
  - - *“At one point, coverage rates declined significantly and with the help of our international partners (NGOs), we implemented… outreach strategies. We went to homes to identify children to be vaccinated. Before, we had mobile teams throughout the department. But since the funding was not renewed we stopped.” (Health worker at the operational level, IS25)*
- Lack of fuel and vehicles:
  - - *“We had mobile teams but we have to face the problem of means: no vehicles, no fuel.” (Health worker at the operational level, IS15)*
- Lack of availability of families due, among others, to distance (living in peri-urban or rural areas), membership in “religious sects”, farming lifestyle (leaving no time to attend vaccination sessions), and/or population movement (e.g., semi-transhumance among pastoralists):
  - - *“The area is too large. In the town there are no problems, but we have problems with people who move a lot. It depends on the season. And there is no infrastructure to organize advanced strategies. The department is large, you can walk for miles and see nobody. You come and you'll see that they are gone. Some of them go back to the center, but it is because they are sick. They come with families, baggage, and sometimes-even cattle. We do not have the resources for mobile and advanced strategies and not even for immunization campaigns. These activities are only done when they are funded by financial partners.” (Health worker at the operational level, IS23)*
- Lack of funding for immunization specifically: Health workers may try to combine funded activities (nutritional campaigns) with immunization activities to overcome funding challenges. Some lay health workers try to improvise “catch-up” sessions. However, they encounter several difficulties including:
- Lack of funding for public transportation: They have to walk and carry supplies (boxes with vials and syringes), which they described as quite heavy:
  - - *“During campaigns or mobile and outreach activities, we carry coolers and sheets, and it's heavy. I didn’t participate in the last campaign because we walk a lot and it is loaded. I walked around the neighborhood but I can’t do it anymore” (lay health worker at the operational level IS21)*

*Refusal attitudes of families*

Health staff described difficulty convincing families with “refusal attitudes” (i.e., opposition to vaccination) to get vaccinated, especially with OPV during campaigns. They proposed various explanations, summarized here:

- The “culturalist” or “ethnic” explanatory model: All health staff identified particular ethnic groups to be more likely to refuse vaccination. These groups are not the same in all health facilities. In general, they are local “minorities”, which are distinct from the health staff’s own group.
- The “religious” explanatory model: Most Senegalese are Muslim, and some belong to smaller Islamic branches. Health workers define these groups as “sects” with roots in Nigeria:
  - - *“The X^^[[5]](#footnote-5)^^ refuse. Women are veiled. All vaccines are refused. Well, there may be exceptions including a woman [who is in charge of a] school. She expects her children and students to be vaccinated.” (Community representative involved in social mobilization, CR3)*
- The “gender” explanatory model: Female health staff considered that fathers are responsible for the refusal of vaccination.
  - - *“The husbands refuse. Sometimes the women say that they would like to vaccinate their child”. (Community representative involved in social mobilization, CR3)*
- “Social class” explanatory model: Upper class or “intellectuals”, including civil servants from the health system, prefer using vaccines from the private sector; and sometimes the richest families in the peri-urban and rural area wait for the immunization of the poorest children to see if there are any AEFIs before bringing their own children. (Community representatives also provide this explanation.)
  - - *“Those who live in residential areas do not trust the EPI vaccines and prefer to take their children to private practices. During the campaign, when we go there, they refuse to show their children, saying they are already vaccinated” (Health worker at operational level, IS19).*
    - *“Educated women first look to other women. When they see that the children are well, they bring them to the health facility.” (Community representative, CR1)*
- Despite these attitudes, various actors are sent to discuss with reluctant families. They can be health workers and their supervisors, district health authorities, female leaders, or religious leaders. Yet, it was reported that these visits do not change behaviors.

## Viewpoints of families and community members

Family and community member attitudes towards immunization sessions can be summarized as follows:

- The majority of mothers are satisfied with immunization sessions, even if they have suggestions for how to improve vaccine delivery and/or health staff behavior. In this group, mothers can be classified as those with significant information on vaccines (mothers with education in health studies), and those with some knowledge and interest in specific diseases (because of previous deaths in their families). In general, they are unaware of the content of vaccines.
- Some mothers described experiences of missed opportunities (i.e., showing up at health centers but not being able to have their children vaccinated) and the feeling of wasting their time. Missed opportunities may arise for three reasons: there is no vaccinator available (when the patient arrives before or after the session time); there is no vaccine; the vaccinator refuses to administer the vaccine if the appointment is not respected or the child does not have a vaccination card. Among the unsatisfied mothers, some of them continue to bring their children to immunization sessions while others give up or postpone visits even if they believe in the usefulness of vaccines:
  - - *“I have two children. One had his first vaccine a few weeks after his birth. I do not remember the exact number of months it was but when I returned to the health facility, my child was not vaccinated. I lost the vaccination card, and I did not know I had a meeting every month. When I came, the health workers^^[[6]](#footnote-6)^^ asked me many questions about the birth, the last time I came, etc. I spent almost the whole morning there; for more time than women who arrived after me. It began to exasperate me and I told them what I thought. Finally I left without my child being vaccinated. You go to the health center, the health workers scold you”. (C18).*

Refusal of all vaccines was expressed in three out of 35 families we interviewed. Three other families refused some vaccines (oral vaccine given during at-home campaigns).

## Explanatory variables for postponement or (occasional) refusal of vaccination among families

This section describes the reasons that may lead families to postpone or refuse vaccination. The below factors are seen as determinant:

- - Social-geographical-linguistic proximity or distance between health staff and interviewees: When health staff and families live in the same place and/or come from the same community, parents are more accepting of vaccination. This is driven by social cohesion:
    - *“We live together. What will my neighbors say if I refuse? They will think that I want to make a problem.” (Caretaker, C4)*
  - Feelings of marginalization among minorities when they do not benefit from outreach strategies:
    - *“What I am saying is that we are excluded from Senegal. Even the drilling was built by the Americans. Last year the doctors came. They consulted people and gave medication, but there was no vaccination. The year before, there was a team that vaccinated children but not all of them. And they received only one injection. I think people do not think about us, they do not make the effort to come here.” (Community Representative, CR9)*
    - *“They do not come because there are no roads; everything is deserted. We even suffer from this. We have no school or anything ... But most of the time, mothers and children stay. You show me the vaccine but I'm sure we will not have it. This is the first time and I think this is the last time. They will not come here to vaccinate us”. (Caretaker, C19)*
- Lack of easy access to health facilities (despite perceived interest of vaccination): This is especially the case for families that live far from health facilities, communities with no health facilities in the area whatsoever, families living around health facilities with insufficient human resources, or transient groups. In regards to access, mothers described three particular constraints:
- Time/opportunity: Families have to wait to have time and negotiate with other household members to perform household tasks while absent; some may take advantage of professional business (trips to local markets) to go to health facilities
- Transportation: Paying for transportation or walking for many kilometers because of the lack of outreach activities
- Financing: Preparing a budget for vaccination to pay for the immunization “ticket” (200 FCFA), the vaccination card, and syringe (as needed):
  - - *“Imagine! We must travel about 30km to go to the post. And when you are up there, you are told: "You have to come back tomorrow, there is no vaccine today" or you have to wait for hours. And when we go home we have to go fetch water, prepare the meal and all that... So we do not go back. If they want us to vaccinate our children, we should reopen the health center because they do not come so far. I have seven children, they are not vaccinated. I gave birth here in the village. When the child is born we put small drops of goat milk in the mouth and eyes and it's over.”(Caretaker, C19).*

## Explanatory variables for categorical refusal of vaccines among families

This section presents the reasons why families may systematically refuse vaccination or give up on vaccination. The first two concern perceptions of immunization techniques:

- Opposition to oral vaccine delivery: One father explained his refusal of OPV because of the oral delivery. He refused all oral medicines but accepted curative and preventive injections:
  - - *“I accept vaccination through blood vessels but not by the stomach. This is the difference between my home and the presidential palace. With injection, the vaccine is given where there are white blood cells, just below the gland. If there is a problem, these white blood cells will respond. They will say “Stop! You can’t go inside!” In the stomach, the product is only with the red blood cells. There is no protection. You see, with the injection, the vaccine enters by the door. With the oral vaccine, it goes from the mouth to the stomach. It is like entering by the roof.” (Caretaker, C6)*
- Opposition to vaccine injection: One mother described her refusal of injectable vaccines considering that they are too painful for their benefits. One other mother, with a “give-up” attitude, also shared this view. It is important to mention that this is a minority view, as all other interviewees believed the pain associated with injection was an indicator of their efficiency.
- Belief that vaccines are useless, as children are healthy without them:
  - - *“Most children in the area are not vaccinated and yet they have nothing. So when it is possible they are vaccinated, if it is not possible, nothing is done.” (Community representative, CR9).*
    - *“After the birth of my second child, I did not return to the health facility for vaccination. Since I left the hospital I never went back. My eldest son has been vaccinated once and you see he is healthy. He has nothing.” (Caretaker, C18)*
    - *“My children are not vaccinated but they are healthy while my neighbor’s children are often ill and they get vaccines.” (Caretaker, C12)*
- Belief that vaccines are useless, based on experience of severe illness after vaccination:
  - - *“I have been vaccinated and yet I am disabled so I do not see why I have to vaccinate my children. My children are all great and have no health problems. I do not believe in what they say. I know that the vaccine is useless. It is God who protects but not men. What happened to me…I take it as a destiny but I do not want my children to receive vaccines. I was vaccinated here at the regional hospital and a few months later I could not walk, I could not feel my legs and I was very sick. My mother was told that I will stay like this because I had polio.” (Community representative, CR 17)*
  - Belief that vaccines are only useful for severe diseases, not for perceived common ones:
    - *“If the vaccinator tells me that children must be vaccinated because the government has decided this, even though these diseases are not common here, I refuse. The government doesn’t support us to treat children suffering from malaria! And in this Koranic school, maybe two children per month are struck down by malaria! Instead of these unnecessary vaccines, the government must use the one for malaria.” (Caretaker in charge of a Koranic school, C14)*
  - Related to the above critique of government decisions on vaccines, some interviewees indicated a lack of confidence in the international decision-making process and insufficient transparency of real objectives:
    - *“NGOs and policy makers have crucial contradictions. They say they are working for the health of children, so that children live, and at the same time they are for birth control and contraception. Human beings make progress but they often make mistakes too. We must look at the history and what the Nazis did. There were doctors who believed in doing well... There are no easy answers. What Africa needs is to be emancipated.” (Caretaker, C6)*
  - Concerns about the quality of EPI vaccines:
    - *“I prefer to buy the vaccine at the pharmacy and then we will do the injection at the health center.” (Caretaker, C4)*
  - Belief that oral vaccines are too “easy” to be effective:
    - *“This is an injection and that's good. That's better to heal someone. I do not believe in oral vaccination. You do not know what they are giving to children, and it's too easy. And it is only in this case they come so far to find us. For injections, there is nobody. We need to go to the health facilities. This is the sign that it is better because it is not easy-to-get.” (Community representative, CR8)*

# Implementation strategy: the potential role of lay health workers in Senegal and assessment of current roles

This section may help to inform predictable issues if Uniject^TM^ were to be implemented, especially regarding real vaccinator training and regulation of fears about AEFIs.

## Immunization stakeholder viewpoints

Various immunization stakeholders at central and operational level – including health workers and lay health workers (trained or not) – were interviewed to understand controversies regarding professional status of real vaccinators, and especially the official and informal/practical roles of lay health workers for immunization.

*Lay heath workers as vaccinators*

- - Denial of role of lay health workers: NIP representatives at central level deny what is actually occurring in health facilities. They insist that only doctors and nurses deliver injectable vaccines. Some members of boards of federations (e.g. nurses and midwives) refuse the (official) involvement of lay health workers because of the:
- Unemployment of graduated nurses and midwives
- Necessity of biomedical knowledge to have safe practices, and especially to avoid nerve damage:
  - - *“Nurses are overworked and unable to do anything. But there are serious drawbacks. The injection is a serious act. Even the nurse normally should not inject vaccines, but doctors delegate. So it is even more dangerous when it is someone who is not trained. Intramuscular injection is deep and can lead to damage of the muscles or nerves. The lay health workers do not know what the risks are or what they do. We can see at school what a microbe is, how the human body (is structured) ... they do not know. There should at least be a selection of those who read. And then there is a problem that comes up. You cannot recruit community workers while there are midwives and trained nurses who are unemployed. These are kids and parents who are invested in their training. They should be valued.” (Immunization stakeholders at central level, IS5 and 6)*
  - Agreement to include lay health workers: Other immunization stakeholders, including health program managers at central level with previous experience at operational level, agreed to involve lay health workers in vaccine delivery. Their reasons were the same as those of graduated health workers at local level: lack of time and human resources and experience over the years watching health staff perform vaccinations (as opposed to young health worker graduates). Two graduated health workers with responsibilities shared their own experience when they were young practitioners, with one of them stating:
    - *“I was a district medical officer. You have to see the need for field staff! Sometimes you find only one person to ensure between 20 and 30 programs. If there are no human resources, how do you make it work? There are 12 million people! Imagine malfunctions due to lack of staff! You see it's complicated. I'll give you a clear example. A midwife has sent a letter to the Minister saying it was outrageous to use traditional midwives, as 2,000 graduated midwives are unemployed. But as for these graduated midwives, I do not see them! They are not organized. They do not go on the ground to make proposals. I have needs. I support those who are there. We need qualified people, but if there is no gynecologist? What is the difference between not having a person and involving a person who makes a mistake one time out of two? We must think about the interest of the population. I have seen several examples. In 1988, I did my rural internship for four months. There were two employees for everyday tasks. The doctor was rarely there. Evacuations were not possible: no vehicle, no fuel ... We tried to find a solution. When the center was built, there was a woman who was trained by a Belgian doctor to help in cases of confinement. Thanks to her involvement, costs were saved and obstacles removed. This woman was empowered to perform certain tasks. When I was an intern, there was another employee who worked in the consulting rooms. A patient came in and the diagnosis was complicated. This employee claimed that the patient had tetanus and he was right. Another time it was a community worker who diagnosed a patient with bacterial meningitis without testing. He was there for over 20 years and he is still there. I have great respect for them. They are limited to what they can do and they do it well.” (Immunization stakeholder, IS3)*
  - Additional reasons evoked for including lay health workers as vaccinators included the confidence of families in health staff coming from the same community, speaking the same language, and living in the same area:
    - *“For the people, it is rewarding to have one member of their own community to care of them as most of the time nurses come from elsewhere. Moreover, when there are graduated health workers, the population prefers the lay health workers.” (Health worker at operational level, IS5)*
    - *“Before, I did not agree to empower the lay health workers. When I arrived, I had a problem with the nurse, who said I refused to empower the lay health workers to keep power for myself. But then there was Y and I saw that she had a strong work ethic. She is exemplary. Even nurses do not do as well. And sometimes people prefer her, as she is a member of the community. They have more confidence in her.” (Health worker at operational level, IS12)*
  - Availability of lay health workers: They live near the health facility while graduated health workers can live in a different place. Moreover, lay health workers tend to stay in the same area for long periods of time, whereas graduated health workers may change job locations:
    - *“We made the choice to help lay health workers to be trained as they have been there for years rather than receive graduated people who were trained but did not stay.” (Health worker at the operational level, IS7)*

*Selection criteria for lay health workers at local level*

Interviewees stated various criteria for the recruitment of lay health workers at local level, including:

- Behavior as volunteers
- Human implication and awareness of responsibilities
- Respect for distinction of authorities and competences without professional status usurpation –even if lay health workers are authorized to wear uniforms or to occupy graduated health workers’ offices
- Capacity to distinguish between emergencies and routine situations, and to refer cases
- Capacity to read and write

*Training strategies for lay health workers according to graduated health workers*

Health facility managers shared the key points for training strategy development for lay health workers:

- Training (initial and follow-up) seen as crucial to empower lay health workers
- Initial training given by graduated health workers
- Regular supervision is assured. The health facility manager is available to discuss cases and receive referral cases. Furthermore, the manager sends competent lay health workers to attend specialized training, even if these sessions target graduated health workers.

In addition, health workers suggested a new strategy involving communities (e.g. COGES). In particular, community groups could sponsor education for lay health workers, leading to certification, and ultimately higher wages. This strategy is described as a reward after a long and faithful participation:

- - - *“We look with the community how to fund their training. [If someone has been a] lay health worker for maybe 10 or 20 years, it is natural to help them to have something.” (Health worker at the operational level, IS7)*

## Community member and family viewpoints

*Distinction of health staff by communities (lay vs. graduates)*

Two parents, and more precisely middle-class fathers with knowledge of the health system organization, distinguished graduated health workers from lay health workers. They expressed a preference for having health facility managers deliver vaccines and provide care to their family.

In most cases, parents, unaware of the distinction between lay/graduate health staff, use the status of “doctor” or “nurse” for all agents. In remote areas with no health facilities, parents only expressed the need for health staff. They indicated a preference for doctors or nurses but concluded that any staff would be good:

- - - *“Anyone would be good to vaccinate our children. If it is a doctor or a nurse it is good.” (Caretaker, C20)*

*Definition of the “good” vaccinator by communities*

Parents and community representatives shared common criteria to define a good practitioner/ vaccinator:

- - Being respectful, gentile, and attentive to mothers and children by speaking with both of them with a sweet voice, explaining care gestures, and answering questions:
    - *“We can make the distinction by the way a vaccinator welcomes the child and by the cry of the child. It's simple, everyone asks for Y.” (Caretaker, C2)*
  - Related to gentleness, having a “light hand” versus a “heavy hand”, as the latter can lead to AEFIs, especially nerve damage
    - *“There can be problems with the injection when the child is restless. The child can have an abscess and hobble along. But this is due to the vaccinator. There are those who have a soft hand and others.” (Caretaker, C2)*
    - *“I prefer the old health worker^31^ (male) to vaccinate my children because I don’t trust the young man. He seems too brutal.” (Caretaker, C16)*
  - In addition, participants expressed a preference for health workers who are “good” community members, as indicated by participation in charitable activities and respect for politeness and religious codes:
    - *“They sent me a young woman, bareheaded and with a mini skirt, to tell me that: "I am the state." I put her out. Y, she is a Christian but she speaks and she does well. And she brings on her back an abandoned child who is disabled. She is a good person.” (Caretaker, C14)*

1. According to Crucell the label during the study was not the same as the actual final labeling. [↑](#footnote-ref-1)
2. Avoiding needle recapping is a safety procedure learnt during training. However, interviews revealed that some vaccinators recap needles, despite the security warnings. This may be an issue with Uniject^TM^ if vaccinators have to repeat activation steps. [↑](#footnote-ref-2)
3. This strategy will be more developed in the section focused on lay health workers. This section will also discuss the official/real role of lay health workers in immunization activities and their social status. [↑](#footnote-ref-3)
4. Each interview is coded. Codes are noted for each quotation. [↑](#footnote-ref-4)
5. We didn’t conduct interviews with members of these groups to document their real attitudes towards vaccine and the determinants of their behaviors. Consequently, we choose to avoid relaying “a kind of denunciation” by using X instead of the group name. [↑](#footnote-ref-5)
6. Caretakers rarely make the distinction between graduated and non-graduated health staff. [↑](#footnote-ref-6)
