# Supplementary material for: Acceptability and Feasibility of Delivering Pentavalent Vaccines in a Compact, Prefilled, Autodisable Device in Vietnam and Senegal
Source: PLoS One. 2015 Jul 17;10(7):e0132292. doi: 10.1371/journal.pone.0132292 (PMC4506041; doi:10.1371/journal.pone.0132292)
Supplement: S2 Data — (DOCX) [file pone.0132292.s002.docx]

# Supplementary file 2: Acceptability study results for VIetnam

# Interviewee viewpoints of Uniject^TM^ in Vietnam

## Overview

Among the different types of interviewees, and regardless of their location, ethnic group, or social groups, we obtained varying reactions towards Uniject^TM^, and sometimes in the same interview.

- Positive attitudes, notably as first reactions: Two main arguments are shared to express enthusiasm:
- Trust in innovation (compared to “old” syringes):
  - - *“It’s been widely used in Western countries for quite a long time. I have seen on TV they use this device to give the insulin injection. (…) In my opinion, we have to change. Injection using the existing kind of syringe is out of date. (…) Because it’s modern, it’s easier and more convenient to use. It’s no longer the time when the nurse has to sharpen the needles, the 5, 10, 20 syringes even got calcified when boiling… No, calcium makes them not fit into each other, some syringes cannot be put in because it deviates by several degrees. We still faced this difficulty in 2000. I remember this unit in 1996 to 2000 that still ground needles. I remember in 2000 or before that, my office still had a pressure cooker with 20 holes to put the needles in for boiling and then we would take it around to give injections.” (DB-DBP-1, village health worker for 36 years)*
- Enhancement of safety (against blood-borne diseases) through auto-disable technology and assurance of appropriate quantity through prefilled doses
- Reluctant or even refusal attitudes, characterized mainly by:
- Lack of confidence in innovation
- Non-relevance of the device for the target, i.e., children (device too weak for restless children or needle too big)
- Difficulty to handle / activate the device, to handle the syringe during the injection, and to squeeze the vaccine (argument of vaccinators who tested the device)

## Findings of feasibility/ acceptability studies

Similar to Senegal, responses are again summarized here by topic: design, safety, ease-of-use, efficacy, dosage, and supply chain (regarding Uniject^TM^ device for pentavalent vaccine).

*Design*

- Appearance: According to some interviewees (parents and health workers), the difference in appearance of the Uniject^TM^ syringe may allay the fears of children due to its fun design:
  - - *“It looks like a piece of candy so I think children won’t fear it, provided that they don’t see a needle.” (AG-CM-KA-2.4, Mother)*
    - *“It’s also very convenient since it looks very neat. The current syringe has a needle that makes children scared. This new device looks just like a toy, thus, they will like it more.” (DB-DBP-NT-2.3, Health worker)*
- Length of the whole syringe: Most interviewees perceived the shorter aspect of the syringe as an advantage. The words “smaller” and “easier” were used in particular. Nevertheless, three health workers expressed doubts concerning the ease-of-use or the safety of the device because of its smaller length (after having used the device):
  - - *“This part, actually, it should be a litter longer to hold more easily. When I withdrew it, I felt that it was relatively short compared to my arm’s length. However, if I adjust my hand posture to hold it, I still feel uncomfortable. It would be much easier to hold if it were a little bit longer.” (DB-DBP-TL-1, Health worker)*
    - *“This part (container) needs to be longer. Thus, it will be easier to hold. This syringe is a little short, thus, slightly moving the hand can touch the baby’s skin. That could be avoided if it is longer. This syringe is too small –a big hand could touch the baby’s skin when holding it.” (Ag-KT-1, Health worker)*
- Length of the needle: Only two health workers considered the length and size of the needle as convenient:
  - - *“I think 2.3 cm is appropriate, no problem. A 2-3 month child is at least about 4-5 kg, it is ok. You may find it big and long, but the intramuscular injection process for children is guaranteed to be a minimum level. Only the specialists can tell what the correct size is.” (DB-DBP-1, Health worker)*
- Size of the needle: All other interviewees, both parents and staff, considered that the Uniject^TM^ needle was bigger than the needle of current syringes^[[1]](#footnote-1)^. They spontaneously expressed their surprise after a first look or they shared their impression after having studied the different characteristics of the device. They notably argued for the needle to be adapted for small infants. According to health workers, the overly long needle could lead to refusal attitudes among the mothers:
  - - *“It is quite easy to use. But its needle seems to be a little bit bigger (…). Children don’t know whether it is small or big but their mothers always watch.” (DB-2, Health worker)*
- Risk of harming infants: Caretakers and some health staff shared concerns of the risk of hurting infants with the needle and “breaking” bones:
  - - *“Can the needle be shortened? I think the needle should be appropriately made for the baby. For example, if it is necessary to inject 1 centimeter into baby’s skin, the needle should be 1 centimeter. If we deeply inject in his/her flesh, I feel sorry for him/her. In some case, if injection is too strong, maybe it can touch the baby’s bones.” (Ag-CM-Kt-2.1, Health worker)*
- Aside from the two experienced health workers, the other interviewees expressed the need to change the needle size. This was often the sole disadvantage pointed out by these interviewees.
- Labeling: Only one community representative gave comments about labeling, suggesting the specification of the number of doses for one vaccine

*Safety*

- Syringe preparation: Some health workers described the advantage of the Uniject^TM^ device for their own safety by avoiding the steps to prepare the syringe. The needle is less exposed than with current syringes when drawing up vaccine:
  - - *“It is safer for those who perform the injection in comparison to the auto-disable syringe. Furthermore, we don't need to directly use these covers. Although we should activate the needle before injecting, it is fairly safe to do. Meanwhile, if we use auto-disable syringe, the needle may hurt us.” (DB-2, Health worker)*
- Temperature control: Health workers noted that Uniject^TM^ may address temperature control issues encountered with traditional syringes:
  - - *“It will be packed in a box and then put into a vaccine carrier. So it’s more beneficial as we can take it out of the vaccine carrier and inject it immediately, whereas the old type takes time to pull the vaccine out of the vaccine bottle before doing the injection. When we pull vaccines out of the bottle, the temperature maybe changed. This one is more convenient and the vaccine is also good.” (AG-CM-KT-3, Health worker)*
- Sterilization: Some health workers highlighted sterilization as an advantage. In effect, the non-exposure of the needle before the injection, the activation mechanism with the needle under the cover, and the device’s sterilized package are perceived as an enhancement for child safety.
  - - *“Absolutely pasteurized! If parents have knowledge, they will prefer this, because it’s safe and clean, no bacteria.” (AG-CM-KA-3, Health worker)*
    - *“It can avoid some catastrophes and infections because it reduces the approach time to the air. So, it can be easy to be infected.”(AG-KT-1, Health worker, quotation before demonstration of Uniject^TM^)*
- AEFIs: Caretakers raised the issue of AEFIs, which they attributed mostly to injection efficacy, and sometimes to a child’s weakness
- Blood-borne diseases: Awareness about non-reusable syringe safety was clearly shared by caretakers, community representatives and health staff. The non-reusable characteristics of the device were often pointed out as the first advantage. One community representative described how parents were concerned by this safety question and verify that vaccinators change the needle between children. According to him, the Uniject^TM^ device presents the advantage of making the first use visible, due to the package and the container, which is deformed after the squeezing step:
  - - *“Some people don’t feel safe with the syringe and don’t trust when health workers throw away syringes. However, this new one is obviously entitled not to be used again. (…) As long as it is preserved well, we can feel safe. We can see when it has been used.” (DB-DBP-NT-2.4, Community representative)*
- Fear of injuries during injection: In addition to the risk of “breaking” bones because of the size/length of the needle, the interviewees described other risks. According to them, current syringes and needles can break away during injection into the arm or leg of the child. This explains two types of reactions among i) those who think that Uniject^TM^ could overcome this problem and ii) those who worry that Uniject^TM^ is too breakable to be able to resist restless children:
  - - *“The old type is difficult to use when babies move or turn. This new one is convenient; you just need to squeeze. Moreover, it’s safer and cleaner. When the health workers immunize, the old type would be dislocated but this new one would not be.” (AG-CM-KT-1.8, Grandmother)*
    - *“I prefer the old one because if the child moves the needle can be broken and I’m afraid that it cannot be injected if the child struggles.” (AG-CM-KA-1.3, Mother)*

*Efficacy*

The stated advantages in this category are largely related to time savings:

- All interviewees mentioned the time saved for health workers and caretakers due to the prefilled device:
  - - *“I like it because it saves time when pulling out a vaccine. Both the old and new ones hurt babies. However, it’s more convenient in terms of its time-saving for immunization because with the old one you have to pull the vaccine out, while this one doesn’t require that. It takes less time: on immunization days, when there are many children need immunizing, health workers are able to work faster.” (AG-CM- KA-1.8, Community representative)*
- One health worker, who had described conflicts between villages notably because of lack of time during immunization days to serve all mothers, perceived the potential benefits of the Uniject^TM^ device to save time and avoid frustration:
  - - *“It doesn’t take much time to pull the vaccine out of the vaccine’s containers. (…) We have implemented EPI for many years. With the old one we could not save time and energy like we can with this one. On the EPI days, for example on the 27th, there were many people coming for immunization but we aren’t able to serve them because we had to separate different target groups. However, with this device we can serve both. It will save time and money. The working period can be contracted to 2 days instead of 3 days as usual. We can serve both children and pregnant women from the different villages at the same time. It should be expanded to mother and child immunizations. I greatly appreciate that. It’s such a convenient and useful device.” (AG-CM-KT-3, Health worker)*
- Time saved was also perceived as a means to avoid frightened children, who will have less opportunity to see the needle:
  - - *“I think it is more convenient than the old one because the old one takes more time to fill the drug inside before injecting into babies’ body. It can be injected faster. Babies don’t see that and maybe they’re not afraid.” (AG-CM-KA-1.9, Mother)*
- Some interviewees also believed that the time of injection could be shorter and, in the end, less painful for the children:
  - - *“It’s convenient in the way that children won’t cry too much because it injects very fast.” (DB-DBP-TL-3, Mother)*
- Avoiding fear and extended pain would improve caretaker experiences, as they would not have to keep their children calm before and during the injection:
  - - *“However, compared to the old method, it will be less time-consuming as I will not have to spend time taking vaccines and parents will not have to struggle to hold their children still for vaccination.” (DB-DBP-TL-1, Health worker)*

*Ease-of-use*

Interviewees who had only observed the device conclude that it appeared easier to use. The vaccinators, health workers, and village health workers, who are familiar with injection techniques and gestures, made positive and reluctant observations about the ease of use of Uniject^TM^:

- Preparation: Most interviewees noted the advantages of the prefilled and one-pack syringe in that it avoided preparation steps.
- Activation: Interviewees identified the advantage of activating the syringe while the needle is in its cover. At the same time, they expressed difficulties with the rotation movement, especially with humid / wet hands, a common occurrence due to the Vietnamese climate (the study team also observed this difficulty):
  - - *“It was difficult as we needed to twist strongly. If our hands were wet, we could not twist to turn it out. We have to twist strong and then back, push and then take the cover off. (…) Just need to pay attention to its usage. It’s very easy to learn to use it. The most difficult thing is how to open and join the needle with the body part. The technique of injection is just the same.” (AG-CM-Kt-2, Health worker)*

One health worker added that this gesture hurts the hands and could be a problem during the immunization days as hundred of injections could be delivered in the same day:

- - - *“I think the twisting step needs to be improved. It is a little bit hard. It is ok for my commune due to few children coming for immunization; however, it would be a problem if the vaccination were for 100 children. I only tried 10 times but my hands are tired. This should be softer so that it is easier for me to press.” (DB-DBP-TL-2, Health worker)*
- The tamper seal (original version): Issues raised included the material used for the seal, the removal process (which may lead to small pieces of plastic that may come in contact with a child’s skin, causing a flesh wound), risk of detachment (leading health workers to believe that an unused syringe has been used and to throw it away, increasing wastage), etc.:
  - - *“The best plastic (nylon) should be similar to medical bottles. It has some dots, so it is easy to turn round. This one is very strong, and soft. That means it should be produced with nylon that is easy to break. However, it should carefully consider the connector, avoiding making it too loose (…) I see that these devices are kept tight; however, it should have a dotted line to be opened easier. I think it is hard to unwrap this device because the nylon is too tough. New staff may be afraid of breaking it when they first use them.” (DB-1, Health worker)*
- Drilling the vaccine container (strength): The movement to drill the vaccine container with the needle requires hand strength. One health worker shared the concern that female health workers would not be able to do this step.
- Injection (speed): When injected, the vaccine is firstly perceived as faster, though some difficulties may arise, especially regarding rapid injection:
  - - *“It’s hard to thrust the vaccine into the body. It is very slow. This device is slower than the old syringe.” (DB-DBP-NT-1, Health worker)*
- Injection (strength): Respondents also perceived that strength is necessary to squeeze the vaccine container during injection:
  - - *“The difference is that you need to give a stronger squeeze to inject it compared to the old one. (…) The body part should be softer. This one is hard so we have to perform a strong squeeze to inject the entire drug into the babies’ body (…). It is better that the tip should be softer. This one is too hard for weak people to squeeze. Only strong people can squeeze it.” (AG-CM-Kt-2, Health worker)*
- Injection (general technique): One interviewee summarized how the Uniject^TM^ device impacted his technique of injection:
  - - *“I think this cover should be thinned a little bit so that it can be squeezed more easily. When I squeezed it, I found that the cover was hard and you needed really strong arms to squeeze it. If the cover were softer, vaccination would be easier. I think that it is much heavier than when pressing the normal plunger. Therefore, if possible, the cover should be softened. (…) This device is not very different to a normal syringe. The only difference in technique is that I need to squeeze and stabilize this needle to adjust my hand and finger posture. With the normal syringe, I only need to use both my hands to hold it. With the old syringe one hand was used to puncture the syringe, the other hand to strain and stabilize the child’s skin. With this device, one hand is used to both puncture the syringe and strain, and the other to squeeze; in that way, it’s quite difficult. Just before we used an orange, but in reality, the child would not stay still like that, right? Thus, it will take time before one can use this device well.” (DB-DBP-NT-1, Health worker)*

During this time, the child could struggle. The handle part is too tight according to vaccinators to secure the injection:

- - - *“With these careful instructions, it will be easier to use. However, it’s quite hard without your introduction. [After the demonstration] I still prefer the auto-disable syringes. I don’t feel that it is certain and tight. Handling it is not tight because children sometimes cry or frisk about. The auto-disable syringe is more secure.” (Db-DBP-Nt-2, Health worker)*
- Piston: Two health workers proposed replacing the container by a piston:
  - - *“I think the needle and its angle are fine, but I think the part where we crush it needs to be changed, if the vaccinator doesn’t crush it hard enough, there may be more unused vaccine than expected. I think it should be replaced with a piston.” (DB- DBP-Nt-1, Health worker)*

All interviewees concluded that vaccinators would need training to become familiar with Uniject^TM^. In the end, if some of them expressed a preference for the current auto-disable syringe, most of them expressed their willingness to use Uniject^TM^. One main argument for this intention was the safety of the prefilled syringe.

*Dosage (and efficacy)*

- Automatic measure (advantages): Some respondents stated that the automatic dosage is advantageous in that it avoids human mistakes and ensures that there is sufficient amount of vaccine:
  - - *“This new one contains a correct dose because it is measured by machine whereas we have to pump vaccines by hand, which may not be precise.”(Db-DBP-Nt-1.2, health worker)*
    - *“Each dose is calculated in one dose. Sometimes, in the middle of the process, the immunizers change position and take a little too much vaccine, or a little less that would not be enough for one dose. So I think this one has already estimated the amount of vaccine needed for one dose. It’s really good. Sometimes we don’t take the exact amount for one dose in which case it wouldn’t take effect.” (AG-CM-KA- 2.2, Health worker)*
    - *“For the new one, we don’t have to fill the syringe with vaccine from a bottle, we just need to shake it well before opening it and then conduct the injection (smiles…). For the old one, it took much time to fill the syringe with vaccine because the amount of vaccine was regulated at 0.5 (ml). Many health workers had difficulty filling the syringe with vaccine. Some couldn’t do it. (…) For the syringe, we have to push strongly on the plunger to insert all of the vaccine into the babies’ body through the syringe needle. This is an auto-disable and disposable syringe, so if the syringe is not filled at 0.5 ml of vaccine, we cannot pull the plunger up to take more vaccine and the syringe is rejected.” (DB-DBP-2, Health worker)*
- However, others shared concerns about the amount of vaccine in the container. They asked if the container had enough vaccine because of the residual dose, and one of them added a comment about the cost that this supplement could represent:
  - - *“Is there no more? I am afraid that a little bit of medicine will be lodged in the top of syringe. Has is been calculated for deduction? When you inject it the vaccine is basically pulled all out. Let's say it pulls out 30% of the full amount of the immunization –the cost may be a little bit higher.” (DB-DBP-1, Health worker)*

*Supply chain*

Interviews noted various advantages in regards to vaccine wastage, transportation, and storage:

- - Wastage
    - Transportation: Given that Uniject^TM^ has a plastic vial (not glass), some caretakers and health workers felt that this could allow for less wastage due to breakage during transportation:
    - *“This new one is better, easier to preserve. The old glass tube could be broken over the long distances when moving.” (AG-CM-KA-1.8, community representative)*
    - Vaccine preparation:
    - *“We can skip 2 or 3 steps when using it, which helps to save the drug and needle that could be broken down midway.”(AG-CM-KT-2, Health worker)*
    - Single dose preparation: It was noted that Uniject^TM^ might avoid wastage compared to vaccines in multi-dose vials:
    - *“We can use each dose for each person. Take this one out of the vaccine carrier and do the injection. It is very convenient. Assume that for the other one a vaccine bottle contains another 20 doses. If this afternoon just 10 of them were used, the 10 left would be thrown away, which is very wasteful. It’s really wasteful and inconvenient. In contrast, this new one provides enough for each child who is immunized. One Uniject^TM^ for each child.” (AG-CM-KT-3, Health worker)*
    - Storage: Interviewees noted the possibility to reduce wastage through facilitated cold chain storage during immunization activities:
    - *“It’s compact and will help medical staff because it is easier to use and move. For example, you just take a bunch of this – about a hundred – to be frozen or something. But that one you must put into a bottle, a vial and if you don’t use it up, you must carefully preserve it in a frozen condition during the immunization session. Sometimes we don’t have enough ice to make it cooler; we must throw it away because it’s useless.” (AG-CM-KA-2.2, Health worker)*
  - Transportation and storage:
    - Packing vials / syringes: Interviewees involved in the storage and transportation of vaccines to health facilities noted that an advantage of Uniject^TM^ is that vials and syringes are packaged together; as such, there is no need ensure that there is a corresponding number of vials and syringes in packages (which is the case with ADSs)
    - *“Packing like this is easier to store and transport than the old device. The old devices are also packed, but there are many 5-in-1 doses in a box. We have to estimate the number of doses needed, which means that we usually don’t get all the doses in a box. We put them in a plastic bag. Then we put the nylon in a vaccine carrier.” (DB-DBP-NT-1, Health worker)*
- Volume: Interviewees compared the volume of Uniject^TM^ to that of traditional syringes, noting the implications in terms of storage / transportation (i.e., need for bigger carriers):
  - - *“The volume of this is similar with the syringes. I think the traditional syringes are harder to be disposed of because they also include a nylon packet. Moreover, they have to dispose the vaccine bottle separately. But we need a bigger carrier.” (DB-1, Health worker)*
    - *“I that think previously, syringes and vaccines were kept separately, right? We do not need to preserve syringes. Only vaccine is required. Now, with this device we need to keep it as a whole and it will take more space when we put it in the fridge, right? It is similar as for transportation. It will require bigger boxes. So, I think it is more difficult. But that’s the only disadvantage. This is indeed easier to use.” (Db- DBp-TL-1, Health worker)*
- Volume (cont.): At the same time, interviewees perceived the small size of the Uniject^TM^ device as an advantage, and notably in comparison with prefilled syringes for rabies vaccines, which are in glass and bigger:
  - - *“It is ok if it is small like this. I thought it would be packed like the rabies vaccine. The prefilled rabies vaccine is a little bit bigger than the UnijectTM device. Therefore, it is difficult to transport. If the UnijectTM device is small like this we can put 50 doses of this kind of vaccine in an ice pack. It is quite similar to the old one, the 5-in-1 bottle.”(DB-DBP-2, Health worker)*

Main findings of “Uniject^TM^ device advantages and disadvantages” (Vietnam)

| **Characteristics** | **Pros** | **Cons** |
| --- | --- | --- |
| **Design** | A lure against children’s fears; Smaller might be easier | Needle too big for infants; Suggestion to add information on the label about the number of doses needed in order to inform parents |
| **Safety** | For health workers as the needle stays under cover until the injection; Sterilization of needle assured by non- exposure to prepared vaccine; vaccine not exposed to changes of temperature during preparation; Regarding blood-borne diseases: Non- reusable and first use is visible, which might allay caretakers’ fears | Risk of “breaking” bones because of needle’s length;  Needle can be broken into the arm or leg of restless children as the device is thin;  Tamper seal break might fail, with risk of skin contact; |
| **Ease-of-use** | Time-saving for health staff and caretakers, useful to reduce missed opportunities and local conflicts, and allay children’s fears; | After using the device, difficulty to handle (too short);  Difficulty to activate: rotary movement (needs strength and hurts hand), tamper seal is an obstacle and could hurt the child’s skin;  The movement to drill the vaccine container needs strength;  Difficult to squeeze the whole vaccine. In the end, injection may be slower, resulting in added pain;  Handle too tight to maintain the device in the arm/leg. |
| **Efficacy / Dosage** | Trust in automatic measure | Residual dose in the container: how to be sure that a sufficient dose is administered? |
| **Supply chain** | Reduction in volume and weight of injection materials including safety boxes; quantity of waste (esp. vials) is reduced; plastic materials may avoid breaking of i) glass vials during transport, ii) needles during preparation, iii) doses as with other mono-dose vials, and because of non-exposure of vaccine when opening vial; avoidance of counting correspondence between doses and syringes and different boxes to pack | Increase of volume to be stored in cold chain; need for larger vaccine carriers |

# Current constraints of vaccination during routine and outreach strategies from the perspective of interviewees in Vietnam

Similar to Senegal, viewpoints of local actors about the pros and cons of the Uniject^TM^ device are based upon their experiences of immunization, and especially injection and ADS (what the theory of acceptability calls “utility”). Given the same objective here of identifying how the Uniject^TM^ device could strengthen the EPI and, ultimately, improve immunization coverage, current barriers to access and acceptability of vaccines are described. This analysis is based on viewpoints of i) immunization stakeholders and ii) beneficiaries, caretakers, and community representatives, who, in Vietnam, are part of the health system organization.

## Immunization stakeholder viewpoints

This section provides an overview of immunization stakeholder viewpoints regarding various immunization topics, starting with the vaccine offer.

*Vaccine offer / demand*

In general, respondents indicated that a lack of vaccines leads to missed opportunities. However, they provided few context-specific explanations for this issue.

- Lack of vaccines (general): Some staff from health facilities at village level described the lack of vaccines and anger of families who were unable to get their children vaccinated during two or three sessions. One reason for this was issues with residential addresses, which may not correspond to those accepted at a given health center (for a particular geographic area). This can be related to movement among temporary workers and/or mothers who change address after marriage, and then bring their children home to their natal village to have them vaccinated.
  - - *“It has no difference for indigenous or non-resident people to use immunization service here. We do not know where they live, they come here to visit their mother or father’s homeland…as long as they follow the schedules and have an immunization book to check, we’re willing to serve them. We don’t let someone miss vaccination. It is no problem if they are residents or non-residents. (…) The provincial health center doesn’t provide enough vaccines to districts, so districts cannot provide enough vaccines to communes. Vaccines are not sufficient in general. It doesn’t mean our health center is ignored. I think the provision isn’t as timely as required. It is also not enough. There is a lack of vaccines for some villages. It happens sometimes, not monthly. I think because many non-residents are served before. Many mothers got married far away such as KG but visited home and took immunization here.” (Ag-CM-KT-3, Health worker)*
    - *“Like this morning, that girl wasn’t from TS district but several dozens of kilometers away from here. Now she left; her kid is here but we can’t inject; tomorrow she will come down there and they will resolve it for her. But she was already too far away. Tomorrow her vaccination period is coming, but it’s her father’s house here. Her husband’s house is in T, she said tomorrow she will think about it. It’s fine then. It’s hard, many people are in KA, but have husbands down in T, they come back here to vaccinate. It’s hard for them... They live with their husband already but still come back here… Moreover, when people from somewhere else come, you must still welcome them. Because actually the target they assigned is too high. Those who married in other countries also return here. They stay at their grandparents, and ask for vaccination. We checked but couldn’t find them on the list…Sometimes they gave birth here, then their mothers raise the kids for months and then they come back to the husband. I don’t know about out there, but in South Vietnam women come back to their parents after getting married.” (AG-CM-KA-3, Health worker)*
- Interruptions following the Quinvaxem incident: During the crisis following deaths linked to Quinvaxem in the media, injections with pentavalent vaccine were interrupted for safety reasons. On the fixed immunization day, health workers only gave oral polio vaccines. As a result, there was a stock-out of OPV for the following immunization day. The apparent motivation for this strategy was to prevent suspicion and questions from families who might have shown up to health centers for vaccination but not received anything:
  - - *“Last month, on the first day of immunization, injected vaccines and oral vaccines were provided for babies as usual but on the second day, we stopped providing injected vaccines because we were asked to do so by the district health center. However, mothers who were invited to the immunization program still took their babies for vaccination so we provided the babies with oral vaccine only. It was asked to stop the injected vaccine to check the quality of vaccine. The district health center informed us of some problems. We still had vaccine for immunization. We did not tell the mothers that we had run out of vaccine; we just told them that the injected vaccine was not provided for this month. We still provided oral vaccines for all babies who were taken here. This month we did not have enough oral vaccines for all babies, so some of them are not immunized with oral vaccine.” (Ag-CM-KT-2, Health worker)*
- This lack of vaccines can lead to irregularity in the presence of families and to conflicts between villages:
  - - *“Vaccines weren’t delivered sufficiently. For example, there are eight villages divided into two periods: four villages organize EPI on the 25th of the month, four other villages on 26th. On the 27th, pregnant women are vaccinated mostly in the morning. However, the four villages that organized immunization on the 26th usually lack vaccines. Many people are dissatisfied and express their opinions to us. They asked to be vaccinated before the four other villages. I also find it inconvenient for health workers in commune health centers and people. Therefore I request more vaccines for immunization.” (AG-CM-KT-3, Health worker)*
- Lack of neonatal doses: An additional issue noted was the lack of neonatal hepatitis doses, even among families who demand vaccines, because it is unavailable in health facilities. Vaccines are available in low-level public health facilities, in some public regional hospitals, and rarely in private facilities. Children who were born at home, in private practices or in some public hospitals, did not receive the neonatal dose. They are apparently not informed about the neonatal dose and its availability in public health centers.
- Willingness to pay for vaccines: According to health workers, even if some vaccines have to be purchased (e.g., Japanese encephalitis or meningitis vaccines), families are ready to pay for them. Even after the attribution of fatal cases to Quinvaxem by the media, some families chose to pay for the 6-in-1 vaccine (pentavalent + IPV):
  - - *“They also ask for paid medicine like Japanese encephalitis vaccine, brain pus vaccine also requires payment but people still take it.”(AG-CM-KA-3, health worker)*

*Parental availability*

- Limited availability among farmers / laborers: Health workers and village health workers expressed the lack of availability of parents who work as farmers or industrial laborers. Children can go with their parents to another place and miss some immunization days as the parents are too busy to come back for the immunization day or cannot miss a day of work:
  - - *“They skip some injections just because some are so poor so they have to work for somebody and they take their children with them while others are too busy with their farming. They don’t have enough time to take their children for immunization. Their children missed some vaccination doses (…). Some work in BD province while others work in GField, which is far from the health station. It is in another commune and in another district also. They leave for work in GField whenever the rice season comes and come back home when the season ends. Therefore, their children have not been vaccinated. They leave for one or two months. They, their husband and their babies, leave. The others including their parents-in-law and older children stay at home to attend school. Their babies are still breastfed so they have to take their babies with them.” (Ag-CM-KT-2, Health worker)*
- Non-respect of immunization calendars: Health workers also noted that grandparents, who may serve as caretakers, might not respect immunization schedules:
  - - *“There is one case where the kid was at home with the grandmother as the parents work in another place. She forgot to bring the child to the health center. Another kid went out with his mother for nearly two years then he came back and lived with his grandparents. In general, children are living with grandparents who do not pay attention to immunization. Thus, the kid is not vaccinated fully.” AG-CM-2, Health worker)*
- Access / distance: Various access / distance issues to vaccination were evoked including the following:
  - Lack of awareness of campaigns: Given that some parents, especially farmers, live far from areas where social mobilization campaigns are conducted (usually on the road using a loud speaker system or in homes by village health workers/female union members or fatherland members), they are often not informed of immunization days:
    - *“Because our radiobroadcast can’t approach field areas, we only hang speakers on or near electricity poles. They have houses here for when they do farming. They grow rice there. There is electricity here but there may not be there. If you got here through the old road, which is being built, you realize it doesn’t have electricity (…) They have to watch their crops. Generally, they don’t have electricity, and therefore don’t notice the propaganda programs. It’s not difficult for us to mobilize them there; however, they are so busy that they don’t pay attention to the programs.” (Ag-CM- KA-2.4, Community representative)*
  - Reach every child: One experienced health worker at village-level described how, in the mid-1990s, vaccines were delivered at-home during the night to be sure to reach every child. He felt that this strategy was effective:
    - *“I even followed my colleagues to give injections. There was no electricity, we just used torches. In the past I remembered that was the diphtheria – pertussis – tetanus injection. We came to one house – the child was sleeping – gave one shot and then went to other houses.” When asked why this was done at night, the same individual said, “Because people went to the field in the daytime. They were not at home.” (DB-DBP-1, Village health worker)*
  - Costs: Another determinant of parental availability for vaccination is access to a vehicle, cost of transportation (fuel for private vehicles, fare for public transportation), and the indirect cost of taking a day off. This is particularly an issue in remote areas and in large districts, but not in districts with a large number of health facilities:
    - *“In my village, the cost of traffic is a problem because the center is quite far from people’s homes. They sometime have to carry their child on their back. They surely come to get vaccinations but they may come late. It takes a whole morning for a return travel. And people here are very poor.” (DB-DBP-TL-3, Community representative)*
    - *“Another difficulty is the distance. In the city, commune health centers are close to households. Moreover, transportation makes the distance less of a bother. But here the commune health center is far away from many families (around 5 – 8km) and they do not have vehicles (…).” (Db-DBN-TL-2.1, Community representative)*
    - *“Some of the women don’t know how to ride a motorbike, then they have to walk to the commune health center while the distance is too long, about 5-6km from here to NN village. The commune is far from the district center.” (Db-DBP-TL-2.3, Community representative*

## Immunization stakeholder viewpoints of parental attitudes

This section describes the views of immunization stakeholders regarding the attitudes of families/community members. According to health workers at all levels, village health workers and community representatives who are in charge of immunization activities and social mobilization, most parents accept and even demand vaccines. Indeed, they are convinced of vaccine efficacy. Discourses can be determined by experience. We can envisage that interviewee responses were influenced by the need to positively present their work and/or the presence of interviewers from the Ministry of Health. However, some interviewees did indeed describe refusal attitudes. Many community representatives shared the questions or doubts of their target populations, while simultaneously describing the success of their mobilization strategies. It should be noted that awareness campaigns are a big part of their work.

- Refusal attitudes: Only a few interviewees described refusal attitudes among parents, with one health worker noting specific challenges among groups in remote areas:
  - - *“People refused us even when we were already at their houses. Two years ago, I was not here, but I was told by my colleagues (one has worked here before me and one has just transferred her work) that ‘It’s very difficult, people here won’t receive vaccination’. After two years working here, I still think it’s very difficult.”(DB-DBP-TL- 1, Health worker at village level)*
    - *“Some groups in remote areas definitively disagree with immunization. They live very far away from the commune health center. We’ve raised opinion on this difficulty in many meetings and training courses. However, we’ve been told that it’s not our problem – because we work in the city that had more favorable conditions than other areas, we don’t talk about it anymore.” (DB-DBP-2, Health worker at district level)*

## Explanatory models of health worker and lay health worker attitudes

- Social acceptance/awareness: One explanatory factor for the attitudes of health staff is the general acceptance of vaccines, awareness of their efficacy, and increasing demand for them.
  - - *“In the past, we did not have immunization programs. For example, with polio we did not have vaccines for such a disease. Some children contracted it and were affected by it and became disabled. At present, there are many kinds of vaccines that can prevent serious diseases so that the people feel secure.” (AG-CM-KT-2, Health worker)*
- Awareness of the cost effectiveness of vaccines through social mobilization
  - - *“People know that they do not waste money for treating diseases, therefore, they do not fall into poverty or near-poverty (…) They are fully vaccinated, they come to vaccinate every month.” (AG-CM-KT-2, Health worker)*
- Confidence in the State/Party
  - - *“If vaccines are free then there is no difficulty. If citizens know about the venue as well as the time for immunization, they are willing come for it because they deeply believe in the health center, the state and the party.” (AG-CM-KA-2.3, Community representative)*
- Reluctance due to fears of side effects: All health workers and lay health workers have described how risks of fever and abscess are well-known by families. Nevertheless, they can temper the enthusiasm for vaccines. It was notably described after the fatal cases attributed to Quinvaxem by media:
  - - *“TV and radio also release some AEFI cases but not in my district. I think it’s due to some exceptions. Generally, it doesn’t matter because mothers are more understandable. They think that is very few cases. It has not happened everywhere.” (DB-DBP-NT-2.4, Health worker)*
    - *“After fever and swelling, the mothers were very worried. They took their children to see me immediately. I told them that I had explained the effects before; that the “5 in one injection” can make their babies feel uncomfortable and cry a lot, thus the caretaker has to follow up with the babies. In some events, caretakers ask why children cannot walk after immunization. I tell them that babies cannot walk because they are in pain. It should last for one day only, and if not, mothers should take their babies to the commune health station for examination. I told them that adults sometimes cannot handle the vaccine for 1 disease, which is why babies need to be injected with a vaccine against 5 diseases.” (AG-CM-KT-2, Health worker)*
- Postponing attitudes: More generally, caretakers from all ethnic groups, in urban and rural areas, can delay immunization if their child presents fever or signs of weakness, for example after premature birth. It is more of a “postponing attitude” for contraindication without interaction with health facilities than a reluctant attitude. Health workers said that this is less likely to be the case among the ethnic majority group (Kinh):
  - - *“Thai people still have unsound customs such as not bringing their children out when they are sick. Therefore, they don’t bring their children for immunization these days. When the children feel better, they will take their children for immunization normally.” (DB-DBN-NT-2.3, Health worker)*
- Negative reactions including crying or AEFIs such as fever may lead parents to avoid immunization when they will have to be available for other activities or to postpone immunization until children are stronger.
  - - *“People in this commune complain about their children falling sick after vaccination. They questioned why their children fell sick after immunization, and said that they will stop getting their children vaccinated. I reckoned that sickness is a good sign, and an expected effect of vaccine. They told me their children cried a lot, and they had to take time off farming work to take care of their children.” (DB-DBP-TL-1, Community representative)*
  - Lack of time/availability or conflicting priorities: Parents may choose to not vaccinate their children for lack of time and/or desire to do other activities. We met two parents with this attitude.

*Refusal attitudes*

Furthermore, some health workers and lay health workers described refusal attitudes in some villages and gave some explanations.

- - Lack of awareness about vaccine efficacy and normal side effects:
    - *“Inhabitants here were very much afraid of vaccination. Their children got sick after being vaccinated, which made them afraid. Methods to change people’s thoughts and behavior should be carried out. Once awareness alters, behavior will change accordingly. Now it is still hard work for local authorities and health workers, as residents here lack self-consciousness of immunization.” (DB-DBP-TL-2.1, Health worker)*
  - Culturalist explanation (Kinh): Based on this theory, there is a problem of awareness among ethnic minorities that can be explained by “habits”
    - *“Most of villagers are Thai people who are civilized and receive a good educational system. Moreover, they have lived with Kinh people for a long time and are integrated with modern customs and habits. Other groups also get along well with each other, they enjoy the knowledge and experience from civilized people. As a result, they’re more well-educated and aware of the effectiveness from the EPI. However, the situation that they worship for feeling better still exists in a small proportion of ethic villagers in the remote area. That is due to their long-standing customs. Nevertheless, they also go to the health center for treatment at the same time.” (DB-DBP-NT-2.1, Health worker)*
    - *“The immunization program was very hard in the first few years. The awareness among Thai, Khmu and Hmong people is different. Khmu people had poor awareness of immunization, Few of them got immunization. Hmong people are now the most difficult in immunization. They still follow backward customs so they don’t get immunization even though we mobilize them (…) For example, they didn’t allow us to go into their homes to inject measles vaccine with a tree in front of their doors, a forbidden gate.” (DB-DBP-TL-2, health worker)*
  - Difference of languages and calendars that limits social mobilization
    - *“Many of them are illiterate and can’t speak Kinh language so it is hard to promote to them. Sometimes they cannot understand what we are saying.” (DB-DBP-TL-2, Health worker)*
    - *“They still remember the dates, but sometimes they are too busy. They just remember the date in moon calendar, not sun calendar.” (AG-KT-2.4, Community representative)*
  - Religious opposition to immunization (the case among some Catholic minorities)
    - *“The deepest reason for refusal relates to many factors such as feelings and community awareness or religions because many people are catholic. To be honest we don't know why; not even we know whether this happens at the grassroots level or not. However, reports have also mentioned the non-cooperation of people. Hmong ethnic group often accept. When we come to vaccinate their children, they still have their child vaccinated. However, only those who live in the nearby areas may accept while the awareness of those who live in the remote and isolated areas is different.” (DB-2, Health worker)*
  - Confidence obstacles: Various confidence issues were reported, including the following:
    - Lack of confidence in the State offer and health staff, as reported by one lay health worker (native) who does social mobilization:
    - *“The majority of people here do not trust the local commune health workers’ ability. They said that the commune staff does not have much experience. (…) The difficulty is that the commune health center is now degraded. When people come here to get vaccination, they see the center and feel worried. The other difficulty is lack of medicine and vaccine (…) People often think that private service is better. They have better vaccines and because there are other benefits, they have better service and make the mothers more satisfied (…) The healthcare center is so crowded.”(AG-CM-KT-2.4, Community representative)*
    - Lack of confidence in vaccinators: Some experienced health workers or village health workers explained the lack of confidence in vaccinators notably due to turnover especially because of training periods:
    - *“I am 22. I am a medical assistant. I have been in charge of immunization activities since March 2012. Everyone has their own job and they have to accomplish their task first. I’m able to immunize but there are enough vaccinators here. When they’re busy, I can help. Maybe I’m not doing injections because I am the new health worker. I work at this post because there were lots of changes in human resources here. (…) Every month, the number of health workers is not stable. They might take leave or go off to study. Therefore, the director of health center has to assign everyone’s task in detail.”(DB-DBP-NT-2, Health worker)*

## Parental viewpoints

This section provides parental / caretaker viewpoints regarding the location of immunization activities and the management of immunization side effects.

- - Location of immunization activities: All caretakers – even those who expressed transportation difficulties – said they prefer immunization activities in health facilities thanks to the screening of the child before and after immunization, and the possibility to ask for nurses or doctors’ advice and help if needed. Some parents linked the description of fatal cases attributed to Quinvaxem in media with this precaution, while the majority of caretakers apparently ignored these events.
  - Management of side effects:
    - Designation of caretaker(s) to calm children: It was reported that the caretaker who succeeds in calming down the children goes to the health facility. Grandparents can also be asked to take care of children until they calm down:
    - *“Actually, my wife takes them to the doctor, because when they are injected, they cry very much and I can’t stop them from crying.” (DB-DBP-TL-3, Father)*
    - *“Both of us decided to immunize the children. But when we came home from work, we saw them crying too much so we didn’t want them vaccinated any more.–With which shot of vaccine was the first child injected? –He was vaccinated 2 times. He cried a lot after the first time, so we didn’t take him for immunization the last two times (…) –He cried convulsively when he received the first vaccines at 2-3 months. We were really worried, so we decided not take him for immunization any more. He got a fever and slept like he was unconsciousness. We were really worried. (…)– What did you do in this circumstance? –We brought him to his grandfather. – Brought him to the grandfather’s house? What did he do? –He couldn’t treat him but we asked him whether he could help.– Did he make him better? Why didn’t you bring your child to the health center? – We had to bring him to his house first; we believed he could deal with that. We didn’t need to go to the health center. –Oh, yeah. Did the grandfather solve it? – My child is getting better now. We brought him to the grandfather’s house this morning. They told us bring our children for vaccination.” (DB-DBP-TL-1.6, a Father)*
    - Other side effects (e.g., fever): Parents explained that they follow the advice of health and village health workers and use cold towels to reduce fever. They also called or visited village health workers when they were far from the health facilities, or the doctor and the nurse when they were neighbors. It is interesting to note that caretakers who had asked for special advice, especially concerning mother and child health, were able to identify a preferred health worker or village health worker and to say that person’s name.

# **Implementation strategy: the potential role of lay health workers in Vietnam and** assessment of current roles

## The organization of the health staff and social mobilization

With the EPI, many actors are involved to supervise or execute immunization activities. In addition to availability of EPI vaccines in public health facilities, EPI BCG and hepatitis B vaccines are available in private health facilities. In parallel with the EPI program, some out-of-pocket vaccines (i.e. Japanese encephalitis and meningitis) can be provided by the injection service at provincial level.

The Preventive Medicine Center at the provincial level supervises human resources and supply needs from districts to communes.

Some discrepancies appeared between the provinces of An Giang and Dien Bien concerning the organization of the health staff. In the districts of An Giang province, the health workers were mainly doctors and medical assistants (persons with medical skills but with no specialization). In commune health centers, the health staff was medical assistants and nurses. In these communes, members of “medical groups” were involved in immunization sessions to provide injections, so long as they have received the training and certificate on immunization, or if they are retired, graduated health workers. In Dien Bien province, in each district two medical assistants supervise EPI activities, but no doctors. At the commune level, medical assistants, nurses and midwives can be involved in immunization activities if they have received the certificate. No retired health workers seem to be involved. Village health workers have apparent limited responsibilities as they do not have medical skills.

Concerning injection practices during immunization session in both provinces, it appeared that nurses do the injection while the medical doctors or doctors do the supervision and screening of the child before and after the injection.

To do the investigation into newborn children and social mobilization, different types of actors can be involved: the “medical groups”, among which are the “collaborators” or “commune volunteers” and the “village health workers”. Depending on the district or commune, these actors may have some medical skills, be retired health workers, or be Commune People’s Committee members, Fatherland members or Women union members (sometimes they double up the status of retired health workers and community representatives). Their availability partly depends on their allowance. In some communes, village health workers receive a better allowance than collaborators, while in other communes it can be the contrary, if commune collaborators are nurses.

## Lay heath workers as vaccinators (health worker viewpoints)

In remote/rural areas, village health workers are mobilized for linguistic or cultural reasons to enhance trust in vaccines and understanding of immunization benefits.

- - - *“It is because people can’t understand the Kinh language that we must coordinate with village health workers in our promotion. However, it is difficult to promote because this is not the village health workers’ duty and their allowance is low so they don’t spend much time helping us.” (DB-DBP-TL-2, Health worker)*

To be allowed to vaccinate, village health workers or health collaborators have to be graduated as nurses or assistant doctors or must have received a certificate of immunization safety from the Pasteur Institute or the Preventive Medicine Center at district level after training in immunization activities (safety and technique), according to the decree “23/2008/QĐ-BYT on the regulation of immunization”.

In other areas, village health workers are not allowed to practice injections on children, despite the fact that many of them have years of experience doing injections on adults, especially during at- home visits. Village health workers shared that they used to vaccinate children in the past. None of them specified the changes in their responsibilities. Nonetheless, they shared concerns about the necessity to have a certificate to inject in case of AEFI, and some of them expressed lack of self-confidence to practice this medical act. A manager from one health center explained these safety issues as follows:

- - - *“I think the village health workers can do this but 100% of district health centers around here don’t allow them to do this. I tell you, many trained village health workers can do injections. They can help the private health services, but public management authorities don’t allow them to do. Because when situations happen, they don’t have the techniques to react.”(DB-DBP-1, Health worker)*

A manager at the local level clarified the issue by saying that if adverse events occur, graduate health workers would be responsible for managing the consequences:

- - - *“The village health workers cannot help us with injections. They only encourage the local people to get vaccinations, and take care of children with high fevers by giving instructions on how to take medicine. We do not allow them to directly give injections. I think in order to have them join in, we need to organize some training classes and get them trained. Only if they are qualified can they join us. Without training I do not think it is a good idea to allow them to give vaccinations. To tell the truth, I won’t let them do injections. If I let them give vaccinations, honestly, with village health workers I am just afraid that they only work part-time, so they can hardly ensure the sterile stage, which can cause abscesses, and it is us that have to deal with the consequences. It will be really difficult then. So I think I cannot be comfortable letting village health workers do the job. They only help us with dissemination. Also in the center, village health workers are not assigned to give vaccinations so we do not dare. In case anything happens, we have to deal with the consequences. Even one case of complication can make it difficult for us to work.” (DB-DBP-TL-1, Health worker*
    - *“I don’t think village health workers or collaborators can use this device because they don’t have a medical background. They just have primary qualifications. (…) We should train them about techniques, positions and safety of injections…there are so many things. (…) Few people working in the medical sector can. Only Mr. C. was a village health worker who did injections, but now there is no one. I think everyone who is trained can use this device but it is not easy because there are so many things that need to be considered, such as pasteurization.”(DB-DBP-TL-2, Health worker)*

The need to be qualified to do an injection is a notion shared by all interviewees at each level of the health system. Some interviewees at central, regional, and sometimes district level believe that lay health workers must be involved in immunization activities as vaccinators, but only if training on Uniject^TM^ devices deals with immunization techniques and safety, and if certificates are provided.

- - - *“Several collaborators are able to use them because they graduated from the medical school. It is necessary to train and grant them the certificate on immunization safety. I really want them to be trained so that they can give me a hand in the expanded program on immunization, because the number of children vaccinated in the village is too large.”(AG-CM-KA-0.2, Health worker)*

However, at operational level, most interviewees affirmed that they do not believe in the abilities of non-graduated staff:

- - - *“They are willing to do it because of their heart, not money. (…) We always select prestigious people to be collaborators, and they are believed widely. (…) In fact, it will be better if they don’t participate in injections because their knowledge is not enough. And their responsibility is limited, thus, we should leave health workers in commune health center to carry out the activity. It is better to not use them in any cases. If we lack staff, we will work for a longer time to meet the requirement.”(AG- KT-1, Health worker)*

According to interviewees, Uniject^TM^ devices cannot overcome the current difficulties involving lay health workers, namely sterilization requirements and the management of side effects. Distinctive roles are justified by this main concern.

## Community member and family viewpoints

*Distinction of health staff by communities*

Caretakers shared their preference for vaccinators with the relevant skills to immunize their children. They believe in the authorities (State or the Party) to hire qualified vaccinators. It should be noted that none of these caretakers described having inquired about training certificates. Furthermore most of them were unaware of the skills of their vaccinators. In addition, most of them didn’t know the name of the vaccinator, except if they were neighbors or had to ask for special advice or interventions. In these cases, the vaccinator or, more often, the lay health worker who does social mobilization became an acquaintance.

*Definition of a “good” vaccinator*

In short, a good vaccinator is a person who is allowed to do the injection. Adjectives used to describe these vaccinators include “enthusiastic”, “gentle”, and “available to give advice and explanations”. When caretakers expressed a preference for a vaccinator, they justified their choice by the fact that they used to be immunized by this person. In parallel, some mothers speaking minority languages expressed a preference for interactions with village health workers speaking their tongue. It is remarkable that all health workers we have met in health facilities are Kinh speakers.

1. Findings are about perceptions. They have to be taken into account as they can influence acceptability. [↑](#footnote-ref-1)
